# AI-assisted discovery of an ethnicity-influenced driver of cell transformation in esophageal and gastroesophageal junction adenocarcinomas

**DOI:** 10.1172/jci.insight.161334

**Published:** 2022-09-22

**Authors:** Pradipta Ghosh, Vinicius J. Campos, Daniella T. Vo, Caitlin Guccione, Vanae Goheen-Holland, Courtney Tindle, Guilherme S. Mazzini, Yudou He, Ludmil B. Alexandrov, Scott M. Lippman, Richard R. Gurski, Soumita Das, Rena Yadlapati, Kit Curtius, Debashis Sahoo

**Affiliations:** 1 Department of Cellular and Molecular Medicine,; 2 Department of Medicine,; 3 HUMANOID Center of Research Excellence, and; 4 Moores Comprehensive Cancer Center, UCSD, La Jolla, California, USA.; 5 Department of Gastrointestinal Surgery, Hospital de Clínicas de Porto Alegre, Porto Alegre, Brazil.; 6 Department of Pediatrics and; 7 Division of Biomedical Informatics, UCSD, La Jolla, California, USA.; 8 Postgraduate Program in Medicine, Surgical Scienceas, Federal University of Rio Grande do Sul, Porto Alegre, Brazil.; 9 Medical School of Federal University of Rio Grande do Sul, Porto Alegre, Brazil.; 10 Department of Pathology and; 11 Department of Computer Science and Engineering, Jacob’s School of Engineering, UCSD, California, La Jolla, USA.

**Keywords:** Gastroenterology, Immunology, Bioinformatics, Cancer, Neutrophils

## Abstract

Although Barrett’s metaplasia of the esophagus (BE) is the only known precursor lesion to esophageal adenocarcinomas (EACs), drivers of cellular transformation in BE remain incompletely understood. We use an artificial intelligence–guided network approach to study EAC initiation and progression. Key predictions are subsequently validated in a human organoid model, in patient-derived biopsy specimens of BE, a case-control study of genomics of BE progression, and in a cross-sectional study of 113 patients with BE and EACs. Our model classified healthy esophagus from BE and BE from EACs in several publicly available gene expression data sets (*n* = 932 samples). The model confirmed that all EACs must originate from BE and pinpointed a CXCL8/IL8↔neutrophil immune microenvironment as a driver of cellular transformation in EACs and gastroesophageal junction adenocarcinomas. This driver is prominent in White individuals but is notably absent in African Americans (AAs). Network-derived gene signatures, independent signatures of neutrophil processes, CXCL8/IL8 expression, and an absolute neutrophil count (ANC) are associated with risk of progression. SNPs associated with changes in ANC by ethnicity (e.g., benign ethnic neutropenia [BEN]) modify that risk. Findings define a racially influenced immunological basis for cell transformation and suggest that BEN in AAs may be a deterrent to BE→EAC progression.

## Graphical abstract

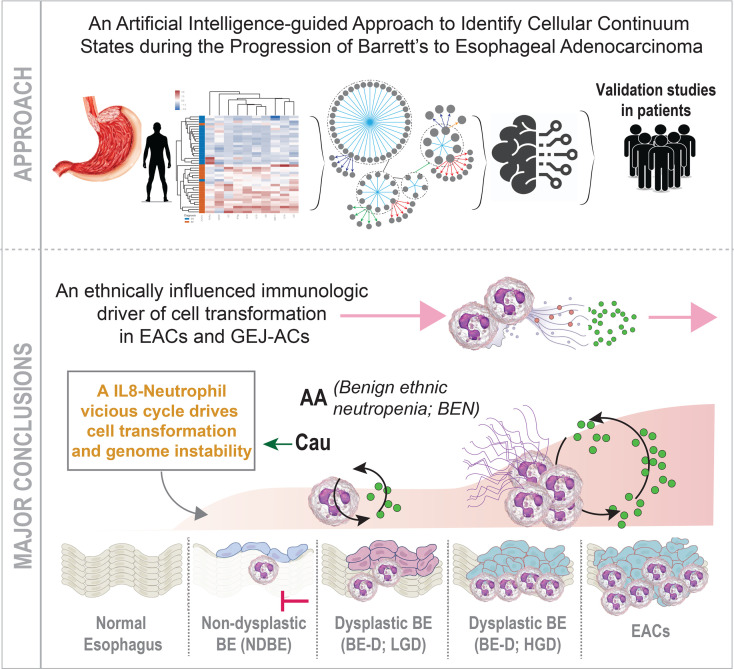

## Introduction

Esophageal adenocarcinomas (EACs) are devastating cancers with high mortality (5% 5-year survival) (1). Barrett’s metaplasia of the esophagus (BE) is the only known precursor lesion; it is not fully understood why only a small proportion of BE lesions progress to EACs (1). Also unknown is why EACs display racial disparity (2); EAC is approximately 4- to 5-fold less likely to develop in African Americans (AAs) than in White individuals. In fact, risk factors for EAC (e.g., long-segment BE and dysplastic BE) are also less frequent in AAs than in White individuals (2).

Although we now know that BE displays early genomic instability and different patterns of progression, these genomic insights are yet to translate into prognostic biomarkers of the risk of BE→EAC progression and answers to fundamental questions (e.g., what drives cellular transformation in BE and if some of those drivers are racially influenced).

**Figure 1:**
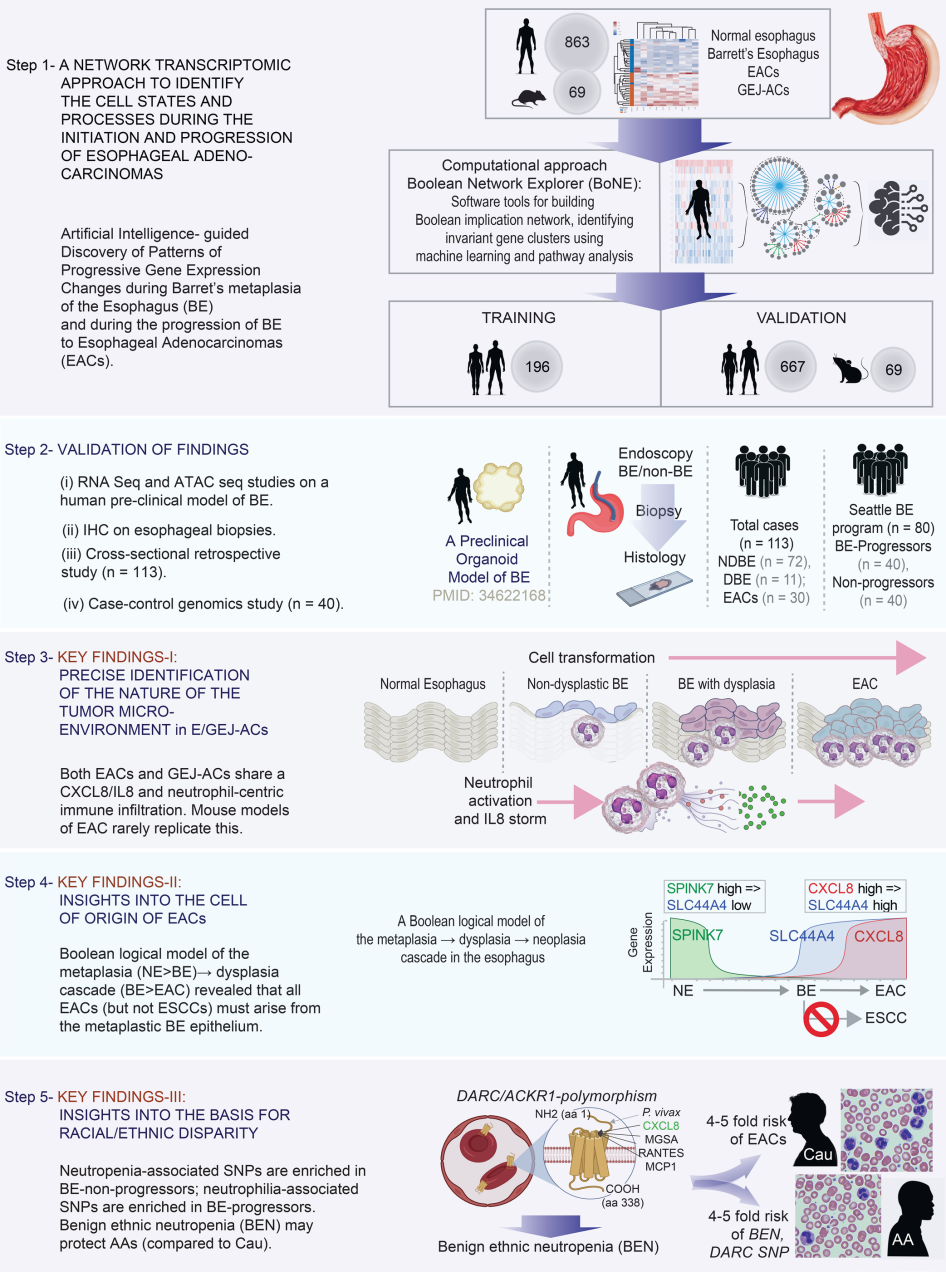
Study design. Step 1: Numerous transcriptomic data sets from both human (*n =* 863) and mouse (*n =* 69) samples were mined to build a validated Boolean implication network–based computational model of disease continuum states during the metaplastic→dysplastic→neoplastic cascade in the squamous epithelial lining of the esophagus. Gene signatures derived from the network-based model are first prioritized by machine-learning approaches and used subsequently to discover cell types and cellular states that fuel the cascade. Step 2: Network predictions were validated in 4 different models and approaches. Steps 3–5: Summary of key conclusions. ATAC seq, assay for transposase-accessible chromatin followed by high-throughput sequencing; EAC/GEJ-AC, esophageal adenocarcinoma/gastroesophageal carcinoma.

To answer these questions, we used a network-based approach involving artificial intelligence (AI) to identify continuum states (of tissues, cell types and processes, and signaling events and pathways) during the process of disease initiation and progression. Modeling human diseases as networks simplifies complex multicellular processes, helps identify patterns in noisy data that humans cannot find, and thereby improves precision in prediction. Once built, the network model can help discover fundamental progressive time-series events underlying complex human diseases and guides the formulation of hypotheses and predictions. We validate key predictions using numerous patient-derived data sets, models, and cohorts to reveal a surprising basis for race- and/or ethnicity-influenced driver of the risk of EAC initiation (Figure 1).

## Results

### An AI-assisted study design.

We chose a Boolean approach to building transcriptomic networks (3); this approach has been used to create maps of evolving cellular states along any disease continuum and identify cellular states in diverse tissues and contexts with high degrees of precision (see *Methods* for details). The Boolean approach relies on invariant relationships that are conserved despite heterogeneity in the samples used for the analysis, which often represent maximum possible diversity (i.e., the relationships can be thought of as general relationships among pairs of genes across all samples irrespective of their origin [normal or disease], laboratories or cohorts, or different perturbations). It is assumed that such “invariants” are likely to be fundamentally important for any given process.

We used the Boolean approach to build maps of continuum states first during metaplastic progression in the normal esophagus (NE) (NE→BE) and, subsequently, during neoplastic transformation of the metaplastic epithelium (BE→EAC). Gene signatures were identified from each map, using machine-learning approaches, and validated in independent cohorts (Figure 1, step 1). Validation studies included using various experimental approaches on human tissues or tissue-derived organoids (Figure 1, step 2). Gene signatures were used as precise and objective tools to navigate new biology and to formulate and rigorously test new hypotheses, which led to a few notable findings (Figure 1, steps 3–5).

### A Boolean map of BE reveals an epigenetic cascade with loss of keratinocyte identity.

**Figure 2:**
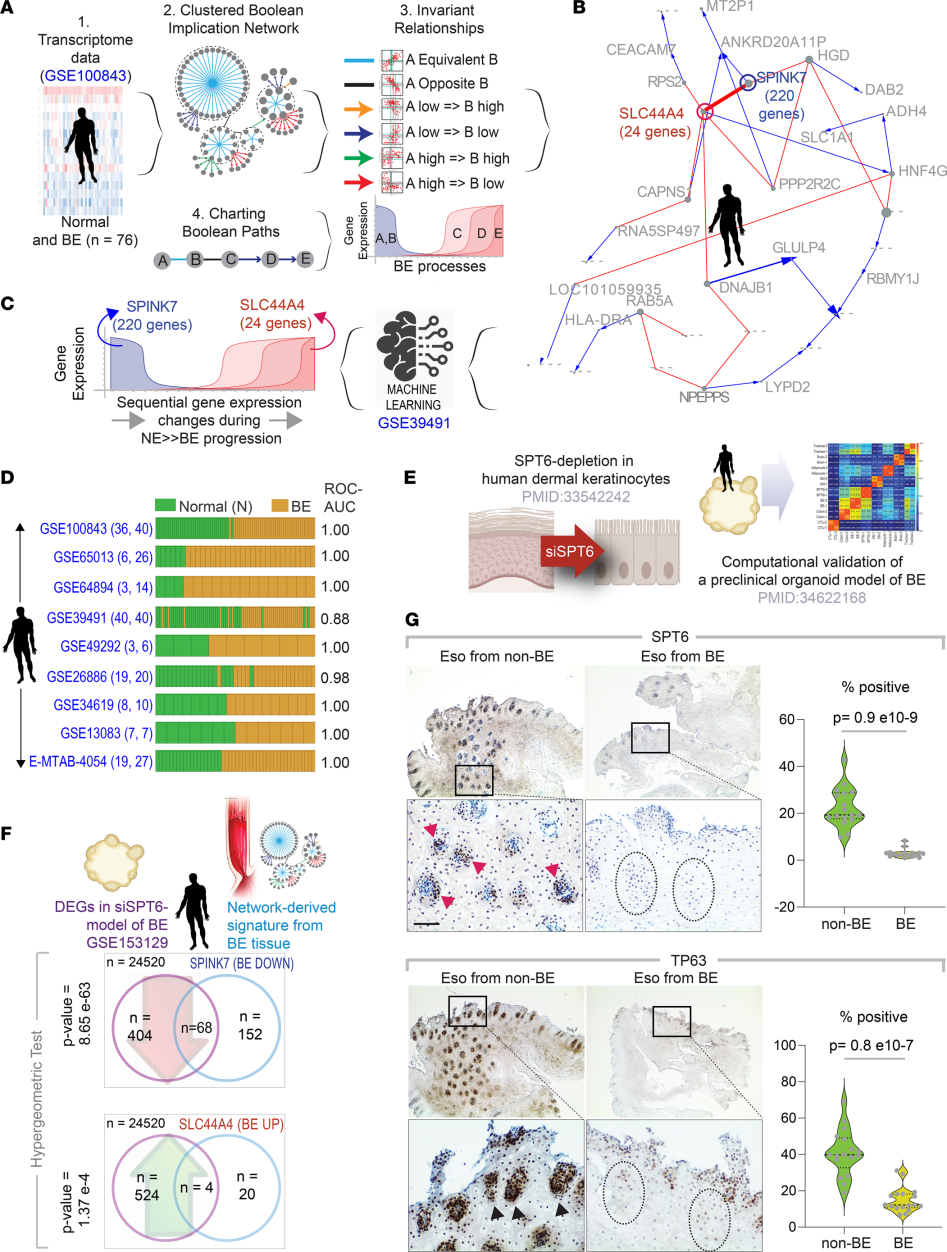
Generation and validation of Boolean network map of BE. (**A**) Schematics outline the workflow (steps 1–4) and training data sets used to create a Boolean map of NE to BE transition using BoNE(4). (**B**) Graph showing invariant patterns of gene expression changes during NE→BE progression. Gene clusters identified by machine learning are indicated in bold. (**C**) Gene clusters in **B** were refined by filtering through a second data set (GSE39491). The resultant signature involves progressive downregulation of *SPINK7* cluster with a concomitant upregulation of *SLC44A4* cluster. (**D**) Bar plots show sample classification accuracy across diverse data sets, with corresponding ROC-AUC values. The sample numbers for healthy (H) and BE analyzed in each data set are annotated on the left margin. (**E** and **F**) Summary (**E**) of a published SPT6-depleted organoid model of BE. Hypergeometric statistical analyses (**F**) show significant overlaps in both up- and downregulated genes between gene signatures identified in the BE maps in **B** and **C** and differentially expressed genes in the SPT6-depleted organoid model of BE (5). (**G**) Esophageal biopsy specimens from men with (Eso from BE) or without (Eso from non-BE) BE were analyzed for SPT6 and TP63 expression by IHC. Red and black arrowheads point to crypts staining positive. Interrupted circles highlight crypts with little or no expression. Fields representative from 3 participants are shown; boxed regions above are magnified below. Scale bar: 100 μm. Violin plots display the percentage of cells positive for staining in regions of interest in **G**, as determined by the ImageJ plug-in, IHC profiler. *P* values were determined by 2-tailed Mann-Whitney test. DEG, differentially expressed gene.

We used Boolean Network Explorer (BoNE) (4) to create a model of progressive gene regulatory events that occur during metaplastic transition (Figure 2A). For model training and development, we used the largest (to our knowledge), well-annotated transcriptomic data set (series GSE100843; *n* = 76) derived from BE and proximal matched normal mucosa from squamous esophagus from 18 patients with BE.

As expanded on in the Supplemental Methods (supplemental material available online with this article; https://doi.org/10.1172/jci.insight.161334DS1), a set of 2 clusters emerged as most robust, and these were further refined by an additional filtering step through a second “training data set” (GSE39491; see Supplemental Methods and Supplemental Table 1) which comprises BE and matched samples of NE from 43 patients. Both training data sets were analyzed independently throughout the process. The resultant model of metaplastic transition pinpointed a time series of BE-associated invariant events in which downregulation of expression of 220 genes (*SPINK7* cluster; Figure 2, B and C) was invariably associated with a concomitant upregulation of 24 genes (*SLC44A4* cluster; Figure 2, B and C) in all samples in the training data sets. The pattern of gene expression signature was sufficient to classify samples in 7 independent validation cohorts and performed consistently well when doing so (receiver operating characteristics AUC [ROC AUC], 0.88–1.00; Figure 2D). A complete list of genes in these clusters and the biological processes that they control (Supplemental Figure 1, A and B) are displayed in Supplemental Table 2. Not surprisingly, the downregulated pathways were enriched for cellular processes that are inherently associated with squamous epithelium.

We found that the network-derived signatures were recapitulated in a recently published organoid model of BE (5) (Figure 2E); that is, overlaps between up- and downregulated differentially expressed genes were significant (*P* = 1.37 × 10^–4^ and 8.65 × 10^–63^, respectively; Figure 2F). This model emerged serendipitously during studies interrogating fate determinants of human keratinocyte stem cells, using an unbiased siRNA screen approach (6) (Supplemental Figure 1C). Loss of transcription elongation factor *SPT6* emerged as a bona fide trigger for epithelial transcommitment from stratified squamous epithelium to a intestine-like lineage, which was attributed to stalled transcription and downregulated expression of *TP63*, the master regulator of keratinocyte fate and differentiation (7). This phenomenon of transcommitment was later shown to faithfully recapitulate the metaplasia-specific signatures of BE, and exposure to acidic pH was sufficient to inhibit the *SPT6*→*TP63* axis in vitro (5).

To determine if the *SPT6*→*TP63* axis is downregulated in the squamous esophageal lining in patients with BE, we prospectively enrolled patients with or without BE presenting for routine care at UCSD and collected biopsy specimens from the distal esophagus, 2 cm above the gastroesophageal junction (GEJ) or the BE segment. IHC studies on FFPE biopsy specimens confirmed that, compared with specimens from participants without BE, both SPT6 and TP63 proteins were significantly suppressed in the esophageal squamous lining from patients whose disease had progressed to BE (*P* = 0.8 × 10^–9^ and 0.9 × 10^–7^, respectively; Figure 2G).

We hypothesized that SPT6 deletion may have altered the genome-wide chromatin accessibility to genes in the *SPINK7/SLC44A4* clusters. Assay for transposase-accessible chromatin followed by high-throughput sequencing studies on the SPT6-depleted BE organoid model confirmed that the downregulated genes in the *SPINK7* cluster (which includes *TP63*) are significantly affected when the histone chaperone SPT6 is depleted. Findings support our prior conclusions (6), in that the *SPT6→TP63* axis maintains keratinocyte identity (pathways enriched in the *SPINK7* cluster; Supplemental Figure 1D) and its loss permits transcommitment or transdifferentiation to a metaplastic intestine-like fate.

### A Boolean map of EAC reveals an immune paradox during cell transformation.

**Figure 3:**
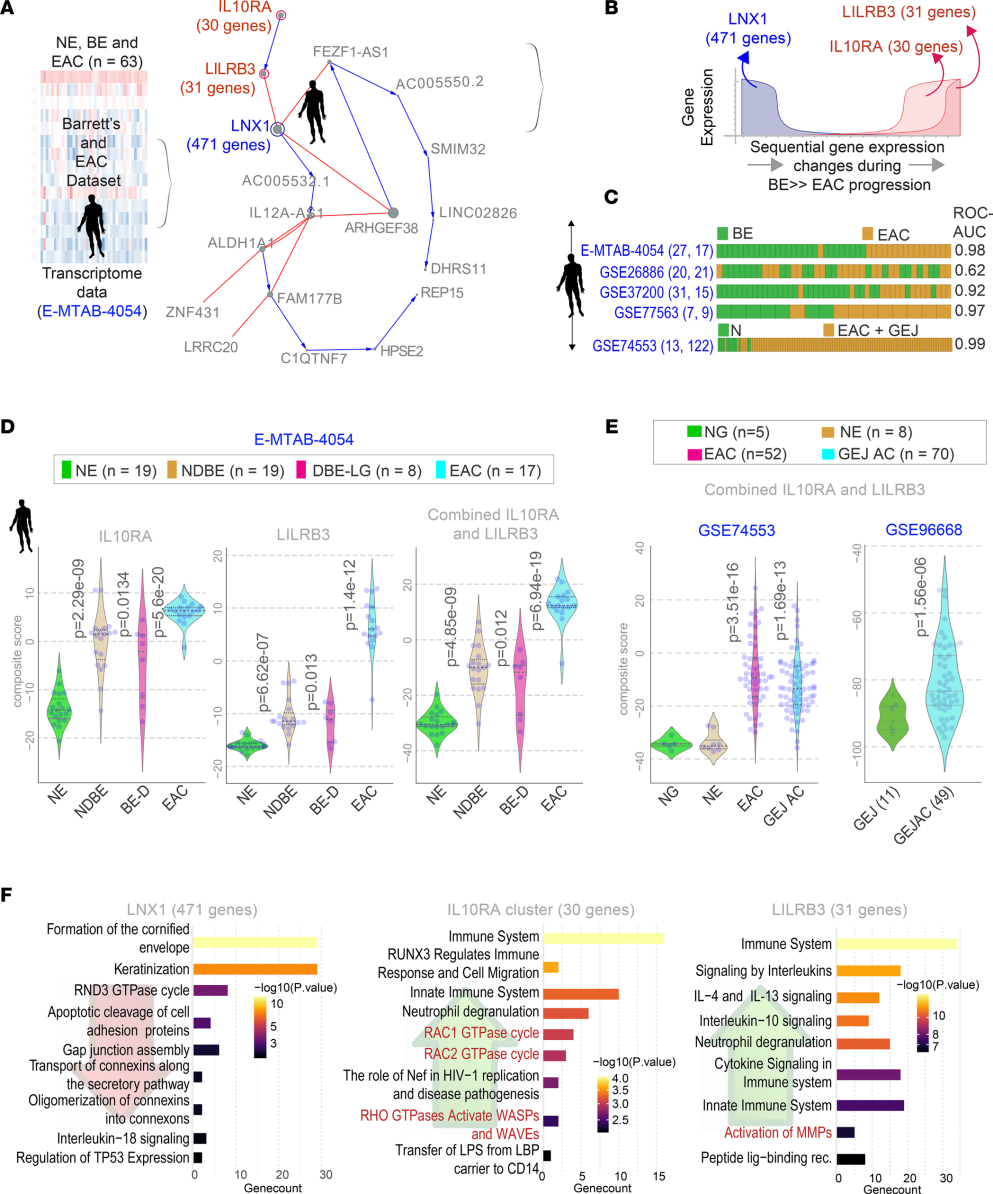
Generation and validation of Boolean network map of BE to EAC progression. (**A**) Schematic outline the workflow and training data sets used to create a Boolean map of BE to EAC transition using BoNE (4). (**B**) Graph showing invariant patterns of gene expression changes during BE→EAC transformation. Gene clusters identified by machine learning are indicated in bold. (**C**) Bar plots show sample classification accuracy across diverse data sets, with corresponding ROC-AUC values. The sample numbers for BE and EAC analyzed in each data set are annotated on the left margin. (**D**) Violin plots show the composite scores of upregulated gene clusters in NE, NDBE, DBE, and EACs. *P* values indicate comparison of each sample type against the NE, as determined by Welch’s *t* test. (**E**) Violin plots show the composite scores of upregulated gene clusters in NE, normal gastric (NG), normal GEJ (GEJ) and GEJ-ACs. *P* values indicate comparison of each sample type against the NE (left) or GEJ (right), as determined by Welch’s *t* test. (**F**) Pathway analyses of gene clusters derived from the map in **B**. Red type indicates likely epithelial processes. DBE-LG, dysplastic BE, low-grade dysplasia; LBP, ligand-binding protein; Lig-binding receptor, ligand-binding receptor; MMP, Matrix metalloproteinase; WASP, Wiskott–Aldrich syndrome protein; WAVE, WASP-family verprolin-homologous protein.

We next created a model of progressive gene changes during BE→EAC transformation (Figure 3A). The following sequence was invariably encountered in all samples: a cluster of 471 genes (*LNX1* cluster) was downregulated, with a concomitant and sequential upregulation of 2 clusters totaling another 61 genes (*IL10RA* and *LILRB3* clusters) (Figure 3A, right, and Figure 3B). Machine-learning approaches pinpointed the *LILRB3* and *IL10RA* clusters as sufficient to classify EACs from BE and to do so reproducibly in 4 independent validation cohorts (Figure 3C). Although the *IL10RA* cluster is upregulated in nondysplastic BE (NDBE) (Figure 3D, left), the *LILRB3* cluster is induced predominantly in EACs (Figure 3D, middle); the composite score of the combined EAC signature shows progressive increase throughout BE→EAC transformation (Figure 3D, right). The genes in these clusters are listed in Supplemental Table 3.

The degree of induction of the EAC signature was indistinguishable in EACs and GEJ adenocarcinomas (GEJ-ACs) (data set GSE74553; Figure 3E, left). This observation was reproducible in another independent data set (GSE96668; *n =* 60; Figure 3E, right).

Reactome pathway analyses of these gene signatures revealed the set of cellular types and states that are progressively gained or lost (Figure 3F). The overwhelming and progressively increasing processes were that of innate reactive immune response and inflammatory cytokine signaling with a predominant neutrophil flare and receptors or ligands that specifically target the neutrophils (e.g., *CXCL8/IL8*, *CXCR1*, *CXCL2*; see the *LILRB3* cluster in Supplemental Table 3) (Figure 3F, middle). These processes are followed by the induction of an immunotolerant or suppressive immune response that is IL-10– and IL-4/IL-13–centric (Figure 3F, right). These paradoxical reactive and tolerant immune responses were associated with a concomitant loss of IL-18 signaling and the TP53 pathway (Figure 3F, left).

### Boolean logic confirms that all EACs must evolve through BE.

**Figure 4:**
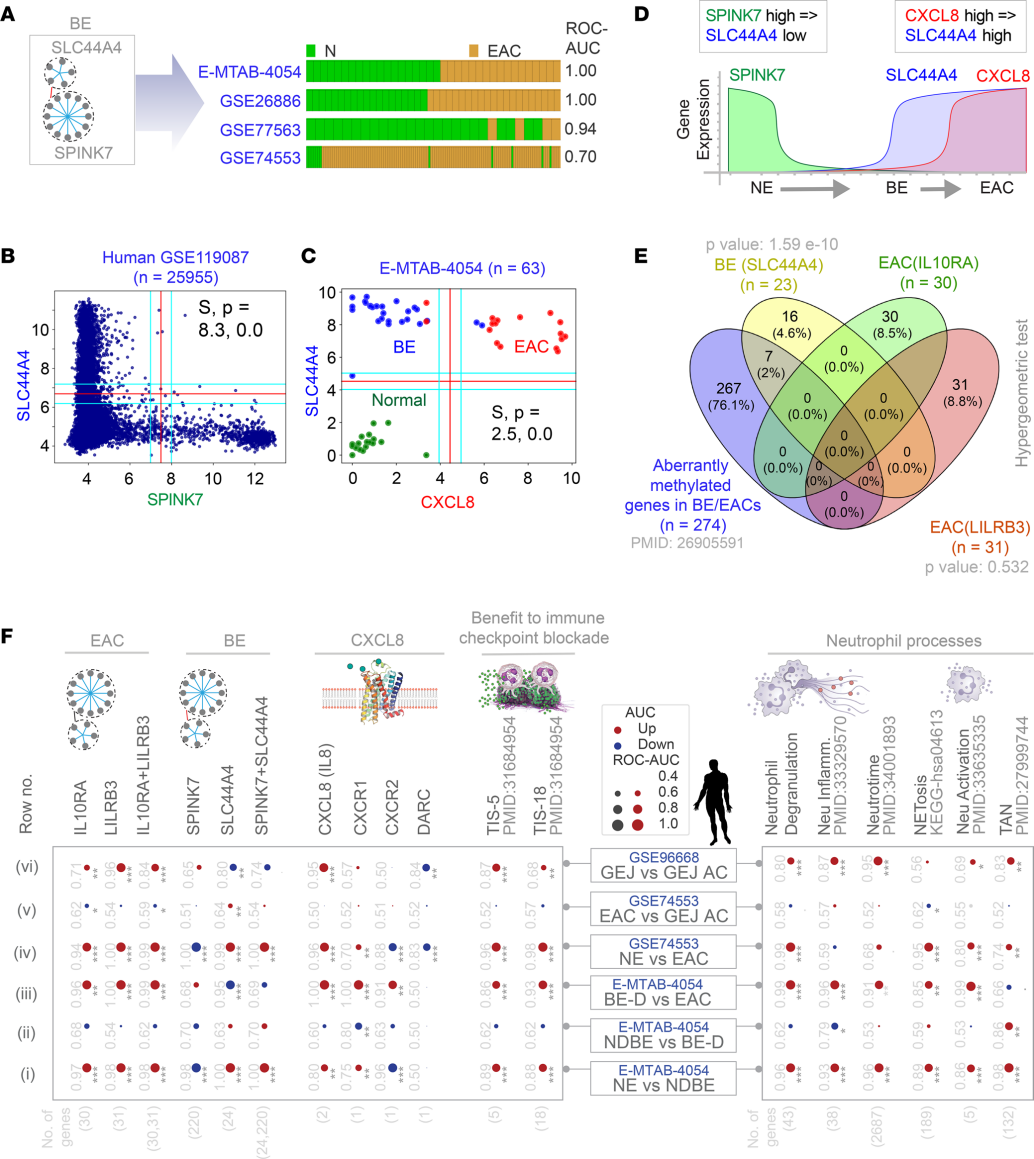
A Boolean logical model of cellular states during NE→BE→EAC progression. (**A**) Bar plots show that BE signatures (Figure 2, B and C) can distinguish NE and EAC samples. (**B**) A scatterplot for *SPINK7* and *SLC44A4* expression in the global human GSE119087 (*N =* 25,955) data set. Boolean implication *SPINK7*-high => *SLC44A7*-low (S = 8.3; *P =* 0.0; FDR < 0.001) is an invariant relationship in the most diverse data set. (**C**) A scatterplot of *CXCL8* and *SLC44A4* expression in the E-MTAB-4054 data set. Boolean implication *CXCL8-*high => *SLC44A4*-high (S = 2.5; *P =* 0.0; FDR <0.001) is an invariant relationship in NE, BE, and EAC samples. (**D**) A schematic to visualize the mathematical model of NE→BE→EAC progression based on MiDReG analysis using BIRs. The model suggests that BE (*SLC44A4* high, *CXCL8* low) must precede EAC (*CXCL8* high, *SLC44A4* high). (**E**) Venn diagram shows the overlaps between the gene clusters from the BE and EAC maps with the genes reported to be methylated in multiple independent studies (*n =* 274 of 22,178 genes tested in total). Only significant *P* values, as determined using hypergeometric analyses, are displayed. (**F**) The human EAC immune microenvironment is visualized as bubble plots of ROC-AUC values (radius of circles are based on the ROC-AUC) demonstrating the direction of gene regulation (upregulation, red; downregulation, blue) for the classification of samples (gene signatures in columns; data set and sample comparison in rows). *P* values based on Welch’s *t* test (of composite score of gene expression values) are provided using standard code (*P ≤ 0.05, **P ≤ 0.01, ***P ≤ 0.001) next to the ROC-AUC. Left: Panel displays the classification of NE, NDBE, DBE, EAC, and GEJ-AC based on the indicated gene signatures (top) in 2 independent data sets (E-MTAB-4054, GSE74553). Right: Panel displays the classification of the same samples based on neutrophil signatures. Violin plots for selected neutrophil signatures are displayed in Supplemental Figure 5.

We used the concept of Boolean invariant logic to create a model that captures first the metaplastic and then the transformation steps of cellular continuum states. First, we found that BE signatures (Figure 2B) are also induced in the EAC samples across diverse cohorts (Figure 4A). The Boolean implication *SPINK7* high => *SLC44A7* low, which defines metaplastic transition in the normal epithelium (Figure 2B), is an invariant relationship in the most diverse global human data set (i.e., GSE119087; Figure 4B), suggesting that this pattern may be fundamentally important. Using *SLC44A4* as a seed gene in a data set that comprised NE, BE, and EAC samples, we found that *SLC44A4* shares an invariant relationship with 1 of the genes in the *LILRB3* cluster, *CXCL8*. *CXCL8* high => *SLC44A4* high is an invariant Boolean implication relationship (BIR) in NE, BE, and EAC samples, where each sample type is mostly confined to 1 quadrant (Figure 4C). This model suggests that if EACs must originate from the esophagus, they must do so via the metaplastic BE intermediate; only 3 genes could nearly accurately classify the samples (Figure 4C) and show progressive expression changes along the continuum (Figure 4D). These findings were validated in a second cohort pooled from multiple independent data sets (Supplemental Figure 2A). By contrast, esophageal squamous cell carcinomas (ESCCs; *n* > 400 samples pooled) did not conform to the Boolean logic–based model of the BE→EC continuum and, as expected, the model confirms that ESCCs do not transition through metaplastic BE states (Supplemental Figure 2B).

Prior work had reported that 274 genes are aberrantly methylated, and indistinguishably so, in 6 independent EAC and/or BE methylation studies (8). Significant overlaps were seen between those 274 genes and the BE-associated *SLC44A4* cluster (*P* = 1.59 × 10^–10^), but not the EAC-associated *LILRB3*- and *IL10RA*-clusters (Figure 4E), suggesting that the methylome in EAC is imprinted early during evolution through BE.

### Two waves of an IL-8↔neutrophil-centric inflammation is encountered during cell transformation.

We noted that the *LILRB3* cluster contained the cytokine *CXCL8* (henceforth, IL-8) and its receptor, *CXCR1*. Discovered as the first chemokine activator of neutrophils, IL-8 displays a distinct target specificity for neutrophils, with only weak effects on other blood cells (9). The *LILRB3* cluster also contained *CXCL2*; the *CXCL2*-*CXCR2* axis helps in the recruitment of tumor-associated neutrophils (TANs) (10). We asked how this IL-8↔neutrophil-centric inflammation varies during the metaplasia–dysplasia–neoplasia cascade by comparing pairwise each sequential step (i.e., NE vs. NDBE; NDBE vs. dysplastic BE [BE-D]; BE-D vs. EAC). The BE and EAC map-derived signatures, as well as IL-8 and its 2 signaling receptors (CXCR1/2) (Figure 4F, left) and numerous pathologic neutrophil processes (Figure 4F, right; Supplemental Figure 3) were significantly induced in EACs (Figure 4F, row iv) and in GEJ-ACs (Figure 4F, row vi). The patterns of induction of all the gene signatures (see Supplemental Table 4 for gene lists) were virtually indistinguishable between EACs and GEJ-ACs (Figure 4F, row v). Upregulation was observed in 2 phases: early during metaplastic transformation from NE to NDBE (Figure 4F, row i) and later during transformation from BE-D to EACs (Figure 4F, row iii) but not during transformation from BE to BE-D (Figure 4F, row ii) (see also Supplemental Figure 3 for violin plots). These results show that the normal→metaplasia→dysplasia→neoplasia cascade is associated with a staircase waveform of IL-8 and neutrophil processes.

### Gene signatures reveal a protumor neutrophilic immune microenvironment.

We next asked if the increased neutrophil processes were reflective of immunostimulating (antitumor) or immunosuppressive (protumor) TANs. To this end, we analyzed a TAN (11) signature that measures the protumor N2 TANs. Both EACs (Figure 4F, far right, last column, row iv) and GEJ-ACs (Figure 4F, far right, last column, row vi) were associated with an induction of TAN signature. Similarly, upregulation in TAN signature was noted in pairwise comparisons of NE versus NDBE (Figure 4F, far right, last column, row i) and NDBE versus DBE (Figure 4F, far right, last column, row ii). ROC AUC and *P* values are displayed for each pairwise comparison in Figure 4F (right).

The induction of protumorigenic TAN signatures was associated also with the pan-cancer marker of adaptive immune resistance, an 18-gene tumor inflammation signature (TIS) (12) and its 5-gene subset (Figure 4F, left, last 2 columns), which predicts benefit of anti–PD-1 therapy in various cancers. EAC signatures, neutrophil processes, and TIS signatures positively and strongly correlated across all EAC and GEJ-AC data sets analyzed (*r* range, 0.8–0.99 for TIS vs. EAC signatures; Supplemental Figure 4). These findings suggest that protumorigenic neutrophils may drive adaptive immune resistance. Furthermore, we found that this tumor immune microenvironment was rarely recapitulated in the currently available animal models of BE→EAC transformation (Supplemental Figure 5; see Supplemental Methods for analyses of animal models).

### ANC and neutrophil signatures prognosticate outcome in BE and EAC.

**Figure 5:**
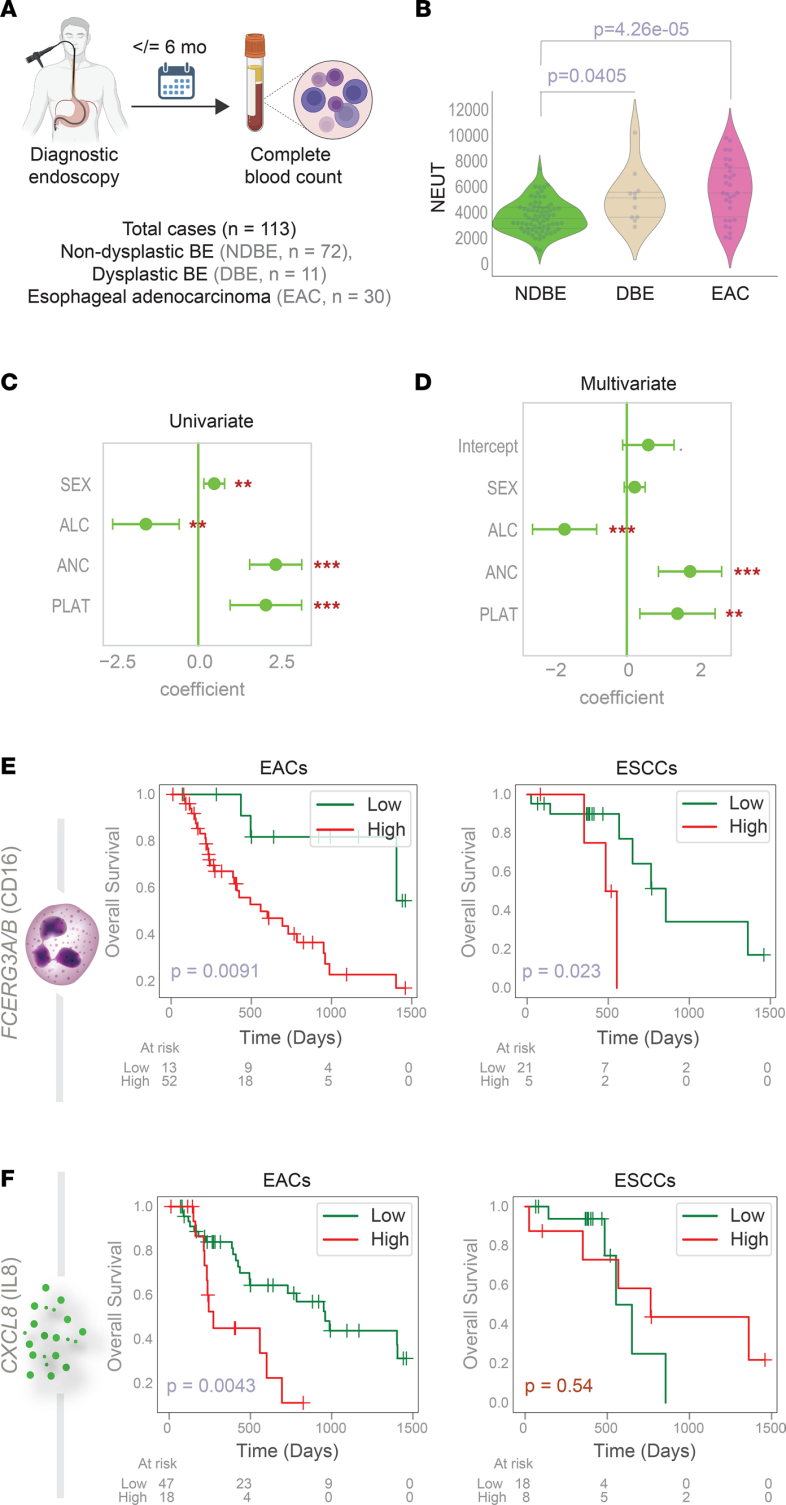
Peripheral neutrophilia and signatures of tumor neutrophil infiltration prognosticate risk of EAC progression. (**A**) Schematic summarizing the cohort composition of a cross-sectional study that is analyzed in panels **B**–**D**. (**B**) Violin plots display the neutrophil (NEUT) counts in various patients within each diagnostic group shown in **A**. *P* values indicate comparison of each subgroup against the NDBE group, as determined by Welch’s *t* test. See Supplemental Figure 6 for other hematologic parameters. (**C** and **D**) Univariate (**C**) and multivariate (**D**) analyses model the risk of BE to EAC progression as a linear combination of sex and the indicated hematologic parameters. Coefficient of each variable (at the center) with 95% CIs (as error bars) and the *P* values are illustrated in the bar plot. The *P* value for each term tests the null hypothesis that the coefficient is equal to zero (no effect). **P ≤ 0.01; ***P ≤ 0.001. (**E** and **F**) Kaplan-Meier plots display the overall survival of patients with tumors stratified based on the high vs. low composite scores of 2 genes (*FCERG3A, FCERG3B)* and the high vs. low expression values of *CXCL8*. *P* values were determined by log-rank analysis. ALC, absolute lymphocyte count. PLAT, platelets.

Next, we retrospectively analyzed a cohort of patients with BE (NDBE, *n =* 72; DBE, *n =* 11) diagnosed between 2013 and 2017 and patients with EACs (*n =* 30) diagnosed between 2005 and 2017 at a tertiary care center in Brazil, with a complete blood cell count within 6 months of diagnostic endoscopy (see Methods for details; Figure 5A and Supplemental Figure 6A). The neutrophil to lymphocyte ratio progressively increased during NDBE→DBE→EAC progression (Supplemental Figure 6B); this was largely driven by increasing ANCs (Figure 5B) and not due to reduced absolute lymphocyte counts (Supplemental Figure 6C). Platelet (Supplemental Figure 6D) and total leukocyte (Supplemental Figure 6E) counts were significantly increased in patients diagnosed with EACs. ANC remained the most significant variable that tracked the risk of NDBE→DBE→EAC progression in both univariate (Figure 5C) and multivariate (Figure 5D) analyses.

Next, we analyzed in EAC data sets curated from The Cancer Genome Atlas the prognostic role of a panel of signatures (Supplemental Figure 7, A–E): the EAC signature, the neutrophil degranulation (signature derived from the EAC cluster), *CXCL8/IL8*, and neutrophil abundance, as estimated in tumor tissues by transcripts of the marker *CD16* (13) (Fc gamma receptor IIIa and IIIb [*FCGR3A/B*]). Although the EAC and neutrophil signatures retained their prognostic impact also in ESCCs (Figure 5E and Supplemental Figure 7, A–E), *CXCL8* did not (Figure 5F). None of these signatures prognosticated outcome in gastric adenocarcinomas (Supplemental Figure 7, A–D, right column).

Findings show that ANC and high intratumoral *CXCL8/IL8* and neutrophil-activation signatures may be disease drivers in EAC and that IL-8–driven neutrophil chemotaxis may be a unique driver in EAC but not in ESCC or gastric adenocarcinomas.

### White individuals, but not AAs, mount IL-8– and neutrophil-centric inflammation.

**Figure 6:**
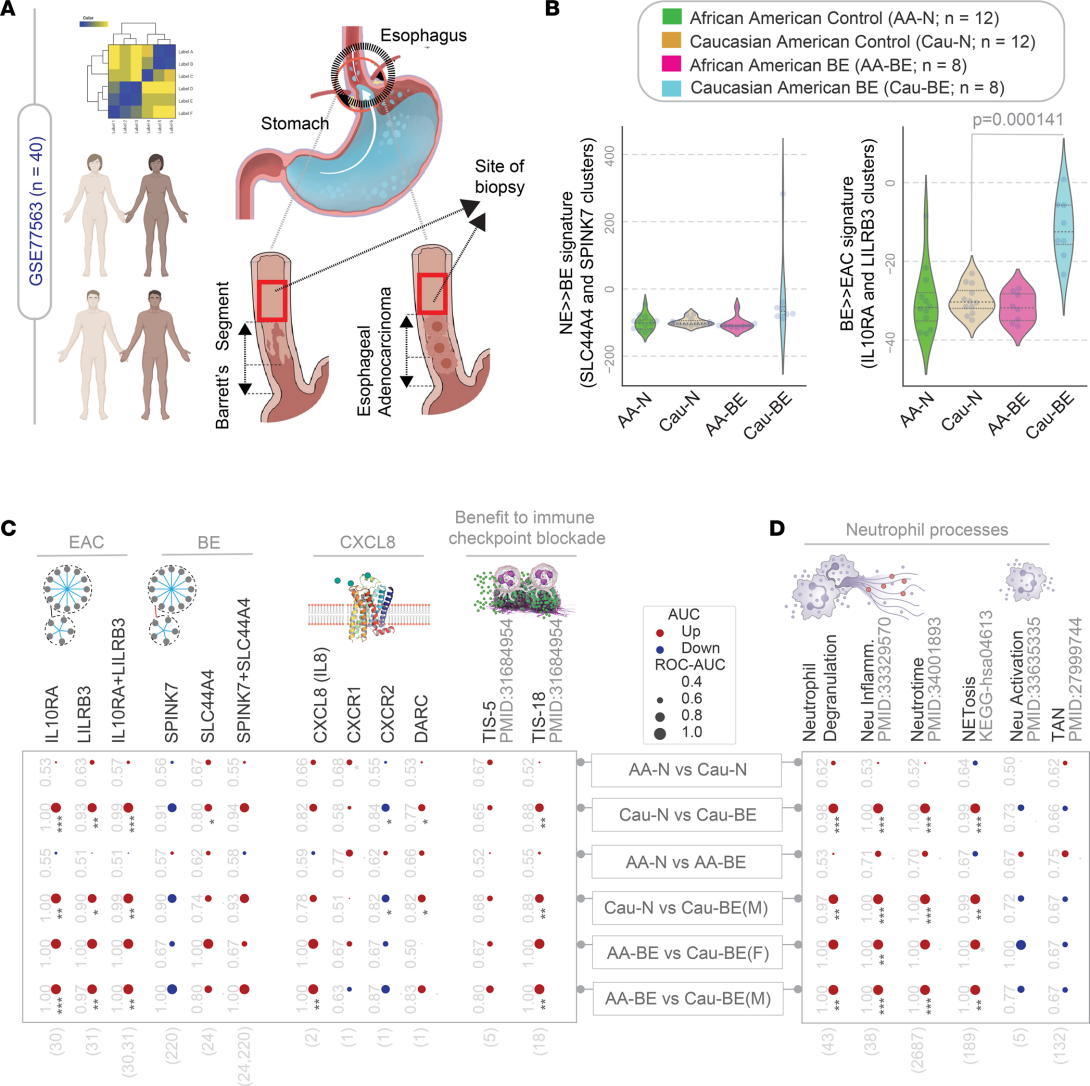
White individuals, but not AAs, mount IL-8– and neutrophil-centric inflammation. (**A**) Schematic displays the study design in data set GSE77563. Microarray studies were conducted on histologically normal squamous mucosa from self-identified AA or White participants, who were healthy (normal control participants), or those diagnosed with BE and/or EAC (AA-BE or White-BE). (**B**) Violin plots showing the composite scores of upregulated gene clusters (*Left*, BE signatures; *Right*, EAC signatures) in control participants (AA-N and White-N) and those diagnosed with BE/EACs (AA-BE and White-BE). *P* values indicate comparison of each sample against the normal samples, as determined by Welch’s *t* test. (**C** and **D**) The human EAC immune microenvironment is visualized as bubble plots of ROC-AUC values (radius of circles are based on the ROC-AUC) demonstrating the direction of gene regulation (upregulation, red; downregulation, blue) for the classification of samples (gene signatures in columns; data set and sample comparison in rows). *P* values (*P ≤ 0.05, **P ≤ 0.01, ***P ≤ =0.001) based on Welch’s *t* test (of composite score of gene expression values) are provided next to the ROC-AUC. (**C**) The classification of AA vs. White samples from control (AA/White-N) or BE/EAC participants (AA/White-BE) in male (M) or female (F) participants are shown based on the indicated gene signatures (top) in GSE77563. (**D**) The classification of same samples in **C** based on neutrophil signatures. Violin plots for selected neutrophil signatures in AA-BE vs. White-BE samples are displayed in Supplemental Figure 8. neu inflamm., neutrophil inflammation.

Next, we leveraged a unique data set containing histologically normal esophageal squamous lining derived from White and AA patients who were either normal (N; i.e., did not have BE or EAC) or were diagnosed with having BE or EAC (data set GSE77563; Figure 6A) to ask how our findings differ along the race or sex divides. Previously, this data set was used to reveal that differential expression of glutathione S-transferase theta 2 (GSTT2), an enzyme that catalyzes the conjugation of reduced glutathione, may protect AAs compared with White individuals from oxidative stress–induced DNA damage (14) (Supplemental Figure 9A). We found that the BE (Figure 6B, left) or EAC (Figure 6B, right) signatures were not different in the squamous lining of the esophagus at baseline (comparing AA-N vs. White-N); however, a diagnosis of BE in White individuals, but not AAs, was associated with an induction of the EAC signatures in the histologically normal proximal squamous lining (Figure 6B, right; compare AA-BE vs. White-BE). When these signatures and all other signatures of tumor microenvironment and neutrophil processes were analyzed systematically, we found that the changes in gene signatures were seen in both sexes (Figure 6, C and D, compare bottom 3 rows; Supplemental Figure 8); however, White men accounted for the most significant changes in the signatures across the board, indicative of progressive inflammation and cell states identified by our network approach. The TIS (12) was induced in White patients with BE, but not AA patients (Figure 6C), suggesting that response to checkpoint inhibitors may differ between the races.

We also noted that (a) *GSTT2* was differentially expressed in AAs compared with White individuals regardless of whether they were healthy or had BE or EAC (Supplemental Figure 9B), and (b) *GSTT2* inversely correlates with the EAC signature (Supplemental Figure 9C) and its subset of neutrophil degranulation signature (Supplemental Figure 9D). Findings suggest that low *GSTT2* (at baseline) and high neutrophil-centric inflammation (in BE and EAC) may synergize as risk factors in White individuals.

### SNPs that increase or decrease ANC are oppositely enriched during BE→EAC progression.

**Figure 7:**
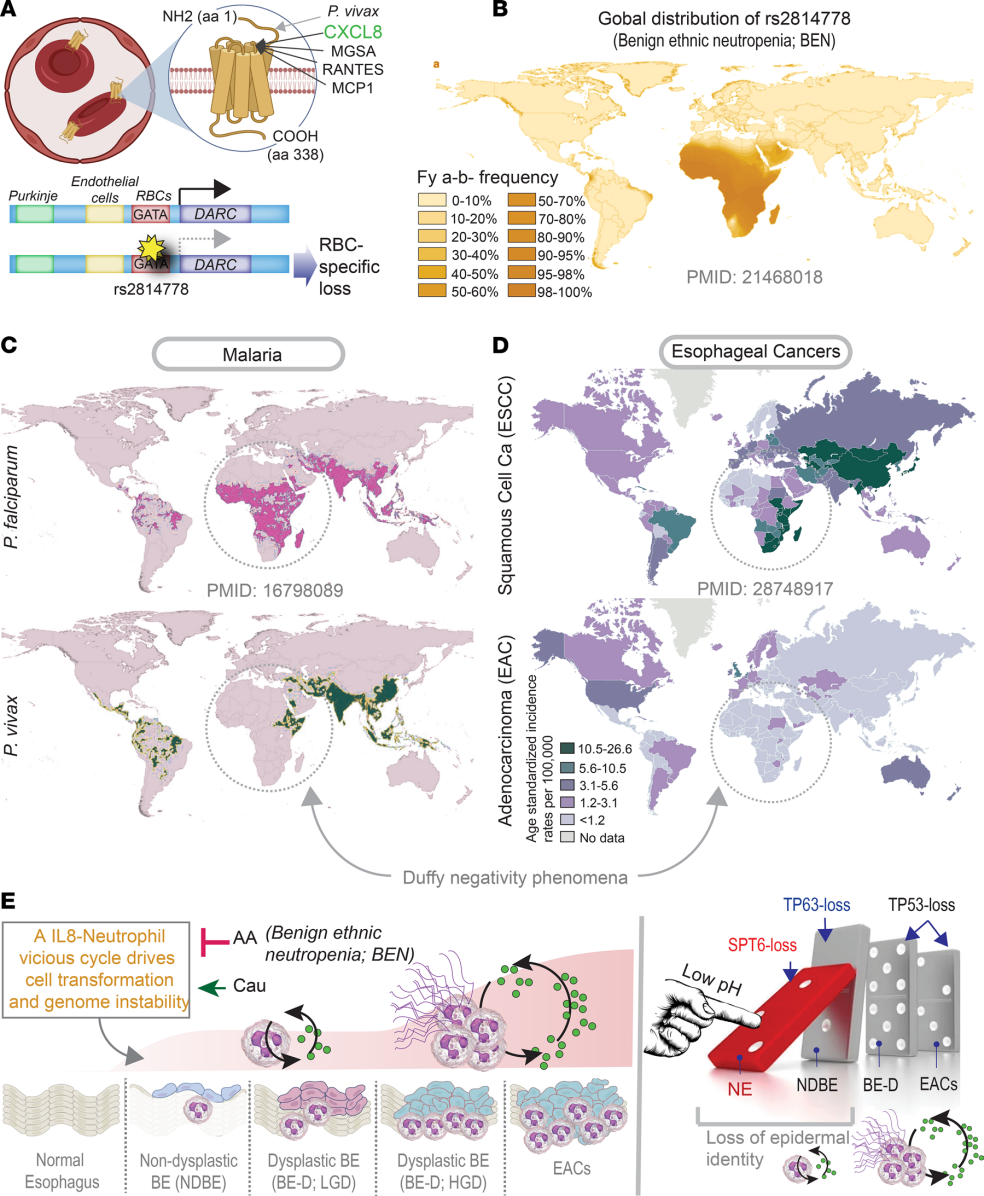
Ethnic neutropenia may reduce the risk of transformation in BE. (**A**) Schematic summarizing the ligands that bind the RBC-localized *DARC/ACKR1* scavenger and the impact of the African polymorphism on RBC-specific loss of *DARC*. (**B**) Global prevalence of the African *Duffy*-null polymorphism that causes BEN. (**C** and **D**) The prevalence of malaria (**C**) and the age-adjusted incidence of esophageal cancers (**D**) are displayed side by side. Black interrupted circles (**C** and **D**) highlight how Africans in *Duffy*-null zones (see **B**) are protected from *Plasmodium* *vivax* (**C**, bottom) but not from *P*. *falciparum* (**C**, top). (**E**) Summary and working model. Left: A vicious IL-8↔neutrophilic storm may be critical for driving the metaplasia–dysplasia cascade during NE→BE→EAC progression. Because of the AA *Duffy*-null polymorphism that manifests as neutropenia and low IL8, some races or ethnicities (e.g., AA, Hispanic/Latino) are protected. Right: As for what permits NE→BE transition, a epithelium intrinsic mechanism may be triggered by suppressed expression of Spt6 in the setting of acid, which, in turn, stalls tp63 function and expression, and a resultant loss in keratinocyte cell fate and gain in metaplastic features. These epithelium-intrinsic mechanisms are likely to be fueled by the vicious IL-8↔neutrophilic storm. HGD, high-grade dysplasia; LGD, low-grade dysplasia.

We next asked if race and/or ethnicity may intersect directly with ANC and the risk of BE→EAC progression. AAs are known to have low ANCs (15), whereas people of Hispanic/Latino descent have high ANCs compared with non-Hispanic White individuals (16). SNPs that either increase (the US Hispanic/Latino population; ref. 17) or decrease (the US AA population; ref. 18) ANCs have been identified. In the case of AAs, the homozygous SNP *rs2814778*, which disrupts a binding site for the GATA1 erythroid transcription factor, resulting in a *ACKR1*-null phenotype (Figure 7A), is known to cause low ANC (18, 19). We used a case-control study (20) that included 80 patients with BE (n = 40 who progressed to EACs and 40 who did not [nonprogressors]); this cohort was selected from a larger case-cohort study within the Seattle Barrett’s Esophagus Program at the Fred Hutchinson Cancer Research Center. Germline data from this cohort were analyzed for the occurrence of 10 SNPs that increase or decrease the ANC, as determined in various studies in the United States (Supplemental Methods). All 3 genes (*DARC/ACKR1*, *ABCC1*, and *HMMR*), influenced by 3 of the 6 tested risk alleles, including *rs2814778* (the allele maximal risk, across studies, and the 1 that confers the risk of BEN; ref. 19) were significantly enriched among nonprogressors, whereas 2 of the 4 protective alleles were significantly enriched among the progressors (Table 1). Among other SNPs that are associated with drug-induced neutropenia in the non-US population, only 1 was significant (i.e., *CYP39A1*; Supplemental Table 6). The opposing patterns of enrichment and de-enrichment of neutropenia-protective and risk alleles, respectively, among BE→EAC progressors was significant (Welch’s *t* test *P =* 0.03978; Table 1). As expected, the frequency of somatic mutations in genes within the EAC clusters (Supplemental Figure 10A) or on genes associated with neutrophil function or number (Supplemental Figure 10B) was higher in BE progressors compared with BE-nonprogressors and tracked tumor mutation burden.

### Ethnic neutropenia may reduce EAC risk.

We noted that the race with the lowest incidence of BE/EAC (i.e., AA), also have the highest incidence of BEN (19), the most common form of neutropenia worldwide. In BEN, a homozygous SNP (*rs2814778*) affects the functions of *DARC* (only in RBCs), which encodes a 7-transmembrane receptor (21) that selectively scavenges inflammatory chemokines (e.g., IL-8 and CCL5, both of which enhance neutrophil recruitment) (Figure 7A). BEN due to *Duffy* polymorphism has explained many mysterious racial disparities in modern medicine (19) but is most prominently known for protecting AAs against *Plasmodium* *vivax* malaria (18). We looked for the *Duffy* negativity phenomenon, which was first described in malaria (22); this phenomenon refers to the geographic distribution of the *Duffy*-negative genotype, *Fy^a–/b–^*, predominantly in sub-Saharan Africa (≥95% Duffy negativity frequency; CI, 75%–95%; Figure 7B), which coincides with the phenotype of near-complete protection from *P*. *vivax* in the same regions (Figure 7C, bottom). It is noteworthy that *Duffy* negativity does not offer protection from *P*. *falciparum* (Figure 7C, top). A strikingly similar contrasting pattern was seen when we compared the global age-adjusted incidence rates of ESCCs (Figure 7D, top) and EACs (Figure 7D, bottom). The African and Saudi Arabian regions, which have the *Duffy*-negative *Fy^a–/b^* genotype (Figure 7B), also have a low incidence of EACs but moderate to high incidence of ESCCs. These findings suggest that BEN could offer selective protection from EACs (just as it does for *P*. *vivax*) in individuals of African descent. Findings also suggest that BEN, which is widely prevalent in other races and ethnicities (e.g., Africans, AAs, Arabs, Yemenite and black Ethiopian Jews, and, to a lesser extent, also in Latinos) but <1% in the non-Hispanic White population in the United States (16), is a possible risk modifier (protective) for BE→EAC progression.

## Discussion

The major discoveries we report here are insights into the cellular continuum states during the metaplasia→dysplasia→neoplasia cascade in EACs and GEJ-ACs, revealed using AI (Figure 7E). Our findings enable us to draw 4 major conclusions, some with immediate and impactful translational relevance.

### The origin of BE and EACs.

Our Boolean logic–based model supports a long-suspected tenet that all EACs arise in BE, which was recently substantiated via multiscale computational modeling studies (23) and through single-cell genomics and lineage tracking studies (24). These 3 approaches independently verify that BE is the invariant precursor to EACs. Our model also confirmed that, unlike EACs, ESCCs do not transition through metaplastic BE states (as expected). TP63 and SPT6 were suppressed in the squamous lining of the esophagus proximal to the BE segment (Figure 2F), and BE/EAC signatures and the neutrophil inflammatory milieu were similarly observed in the normal squamous lining of the esophagus proximal to BE and EAC lesions (Figure 6, A and B), suggesting that the histologically so-called normal esophageal lining is abnormal by all molecular (i.e., protein and gene expression) metrics among patients with BE or EAC. This evidence lends support to a transcommitted esophageal keratinocyte being a cell of origin of BE and EAC/GEJ-ACs, as has been suggested by others (25–28). By showing that the loss of *TP63* occurs early during the BE→EAC continuum, our model captures the key molecular trigger for keratinocyte transcommitment reported previously by others (29). Finally, because key features of the human disease is recapitulated in the IL-1β–*tg* murine model of BE-like tumorigenesis (30) (Supplemental Methods), where the glandular epithelia at the GEJ/cardia gave rise to BE, these glandular cells could serve as an alternative cell of origin of BE and EAC/GEJ-ACs.

### Neutrophilic assault assists cell transformation.

We found that an IL-8–neutrophil-centric immune microenvironment is increased first during metaplastic transition and more prominently later during neoplastic transformation (Figure 7E). This biphasic-wave pattern may explain why long-standing BE carries low risk of EAC (31), presumably because a second wave of immune storm is required for cell transformation. Induction of IL-8 and high neutrophils in BE tissues (32) and circulation (33) has been reported; however, in showing that this immune microenvironment is prominently induced in White individuals but not in AAs, which mirrors the approximately 4- to 5-fold higher risk of EACs in White individuals compared with AAs (34), our findings suggest that this inflammatory microenvironment is likely to be a driver event. We also showed that a high ANC in circulation is an independent determinant of NDBE→DBE→EAC progression and provides epidemiologic evidence that low ANC is a possible risk modifier (protective) for BE→EAC progression in AAs, Arabs, and other races and ethnicities. The fact that peripheral neutrophilia and intratumoral signatures of neutrophil processes are aligned with risk of EAC is not unusual, because such alignment is observed and carries poor prognosis in diverse cancers (35). These insights lend support to the prioritized testing of a class of neutrophil-targeted therapeutics; such drugs could prove beneficial as both single-agent and adjuvant therapy. Because high neutrophil counts with high tumor mutation burden in diverse cancers (including esophageal) is known to reduce the efficacy of the checkpoint inhibitors (36), our results predict that neutrophil-targeted therapeutics may synergize with checkpoint inhibitors.

### Ethnic disparity in EACs/GEJ-ACs may stem from DARC polymorphism.

Our studies shed valuable insights into how *DARC* polymorphism *rs2814778* may shape the risk of EACs. For example, gene expression signatures of neutrophil infiltration and activation are induced in both EACs and ESCCs; however, the existence of a *Duffy*-negativity phenomenon in EACs (i.e., that the *Fy^a–/b–^* genotype is associated with low incidence of EACs, but not ESCCs) suggests that the *Duffy* polymorphism influences EACs through mechanisms other than being the most important genetic determinant of BEN. *DARC* polymorphism *rs2814778* affects serum levels of IL-8 (37). Because EACs, but not ESCCs, significantly induce IL-8, it is possible that the infiltration of TANs in EACs is gradient driven and that gradient is maintained by the RBC-localized scavenger of IL-8, *DARC* (i.e., a cytokine “sink”) (21).

### EACs and GEJ-ACs are similar.

Our study objectively establishes the degree of similarity between EACs and GEJ-ACs at a fundamental molecular level. This finding is in keeping with the fact that, much like EACs, GEJ-ACs are also associated with short and long segments of BE, suggesting that they arise from underlying metaplastic epithelium (38). It is possible that much like EACs, GEJ-ACs evolve through the intestinal metaplastic continuum and that neutrophil-targeted therapies that emerge in EACs are expected to have crossover benefits in GEJ-ACs.

## Methods

Detailed methods for computational modeling, AI-guided prediction and validation, and description of validation models are presented in Supplemental Methods and mentioned in brief here.

### Computational approach.

An overview of the key approaches is shown in Figure 1. Modeling continuum states within the metaplasia→dysplasia→neoplasia cascade were performed using BoNE (4). We created an asymmetric gene expression network, first for metaplastic progression from NE to BE and separately for the dysplastic→neoplastic cascade during BE to EAC progression, using a computational method based on Boolean logic (3). To build the BE/EAC network, we analyzed 2 publicly available transcriptomic data sets (GSE100843 and GSE39491 for BE and E-MTAB-4054 for EACs; Supplemental Table 1). These 2 data sets (our test cohorts) were independently analyzed and the resultant signatures were kept separate from each other at all times. The BoNE computational tool (Supplemental Methods) was introduced, which uses asymmetric properties of BIRs (as in MIDReG algorithm; ref. 3) to model natural progressive time-series changes in major cellular compartments that initiate, propagate, and perpetuate cellular-state change and are likely to be important for BE/EAC progression. BoNE provides an integrated platform for the construction, visualization, and querying of a network of progressive changes much like a disease map (in this case, BE and EAC-maps) in 3 steps. First, the expression levels of all genes in these data sets were converted to binary values (high or low) using the *StepMiner* algorithm (39). Second, gene expression relationships between pairs of genes were classified into 1 of 6 possible BIRs and expressed as Boolean implication statements; 2 symmetric Boolean implications — “equivalent” and “opposite” — are discovered when 2 diagonally opposite sparse quadrants are identified, as well as 4 asymmetric relationships, each corresponding to 1 sparse quadrant. Although conventional symmetric analysis of transcriptomic data sets can recognize the latter 2 relationships, such an approach ignores the former. BooleanNet statistics is used to assess the significance of the BIRs (3). Prior work (4) has revealed how the Boolean approach offers a distinct advantage from currently used conventional computational methods that rely exclusively on symmetric linear relationships from gene expression data (e.g., differential, correlation network, coexpression network, mutual information network, the Bayesian approach). The other advantage of using BIRs is that they are robust to the noise of sample heterogeneity (i.e., healthy, diseased, genotypic, phenotypic, ethnic, interventions, disease severity), and every sample follows the same mathematical equation and hence is likely to be reproducible in independent validation data sets. The heterogeneity of samples in each of the data sets used in this study is highlighted in the Supplemental Methods. Third, genes with similar expression architectures, determined by sharing at least half the equivalences among gene pairs, were grouped into clusters and organized into a network by determining the overwhelming Boolean relationships observed between any 2 clusters. In the resultant Boolean implication network, clusters of genes are the nodes, and the BIR between the clusters are the directed edges; BoNE enables their discovery in an unsupervised way while remaining agnostic to the sample type. All gene expression data sets were visualized using Hierarchical Exploration of Gene Expression Microarrays Online framework (4).

### Data availability.

All data are available in the main text or the supplemental material. Publicly available data sets used are enlisted in the Supplemental Methods. The software codes are publicly available at the following links: https://github.com/sahoo00/BoNE (9def8120b60ec962b2508d5a5c65c9837ed79df9) and https://github.com/sahoo00/Hegemon (51c50b7ae0dff7b76a5e48cef737e17bc141d76f).

### Statistics.

*P* values were computed using the 2-tailed Welch’s *t* test. Gene signature is used to classify sample categories, and the performance of the multiclass classification is measured by ROC-AUC values. A color-coded bar plot is combined with a density plot to visualize the gene signature–based classification. All statistical tests were performed using R, version 3.2.3 (2015-12-10). Standard *t* tests were performed using the Python *scipy.stats.ttest_ind* package (version 0.19.0) with Welch’s 2-sample *t* test (2-tailed, unpaired, unequal variance [equal_var=False], and unequal sample size) parameters. Multiple hypothesis correction was performed by adjusting *P* values with *statsmodels.stats.multitest.multipletests* (fdr_bh: Benjamini–Hochberg principles). The sample number of each analysis is provided with associated plots beside each GSE accession number or sample name. Pathway analysis of gene lists was carried out via the Reactome database. Reactome identifies signaling and metabolic molecules and organizes their relationships into biological pathways and processes. Kaplan-Meier analysis was performed using the Python *lifelines* package, version 0.14.6. The statistical significance of Kaplan-Meier plots was assessed by log-rank test. The Cox proportional-hazard models was used to evaluate the association between the survival time of patients and 1 or more predictor variables. Violin, swarm, and bubble plots were created using the Python *seaborn* package, version 0.10.1. Hypergeometric-tests were performed to evaluate the significance of overlaps between 2 list of genes. Both binomial and Fisher exact tests were performed to test the significance of SNPs. StepMiner analysis (39) was performed to binarize numerical data, and BooleanNet statistic (3) was used to test the significance of BIRs.

### Study approval.

For assessing the impact of ANC on BE/EAC diagnosis and outcome of EACs, we retrospectively analyzed patients with biopsy specimens indicating BE between 2013 and 2017 and with a complete blood cell count within 6 months from the endoscopy, as well as patients with EAC at a tertiary care center in Brazil (Hospital de Clínicas de Porto Alegre). Cases (*n =* 113) were classified as NDBE (*n =* 72), DBE (*n =* 11), and EAC (*n =* 30) (33). The study was approved by the Brazilian National Committee on Research Ethics (registration no. CAAE-81068617.2.0000.5327).

For collecting esophageal biopsy specimens for IHC, we enrolled patients undergoing endoscopies as a part of their routine care and follow-up at UCSD’s Center for Esophageal Diseases. Patients were recruited and consented using a study proposal (project identification no. 200047) approved by the UCSD Human Research Protection Program IRB (project identification no. 200047).

Human keratinocyte-derived BE-like organoids were created, characterized (by morphology and molecular level) (6) and validated computationally (5) previously using an approved IRB (project identification no. 190105) that covers human subject research at the UCSD HUMANOID Center of Research Excellence. For all the deidentified human participants, information including age, sex, and previous history of the disease was collected from the participants’ charts following the rules of Health Insurance Portability and Accountability Act of 1996. The study design and the use of human study participants was conducted in accordance with the criteria set by the Declaration of Helsinki.

## Author contributions

DS and PG conceptualized the project; DTV, under the supervision of PG and DS, carried out computational analyses; DS contributed all software used in this work; RY, VJC, GSM, and RRG provided access to patients and secured all tissue samples used in this study; VGH carried out the immunohistochemical studies under the supervision of PG; CT and SD were responsible for the BE organoid model studies; CG, YH, and KC carried out the mutation and SNP analyses in BE data sets, accessed through the pre-cancer genome atlas (PCGA) pipeline co-created by LBA and SML. DS and PG prepared figures for data visualization and wrote the original draft of the manuscript. All authors provided input, edited and revised the manuscript, and approved the final version of the manuscript. PG coordinated and supervised all parts of the project and administered the project.

## Supplementary Material

Supplemental data

Supplemental data set 1

Supplemental data set 2

Supplemental data set 3

Supplemental data set 4

Supplemental data set 5

Supplemental data set 6

## Figures and Tables

**Table 1:**
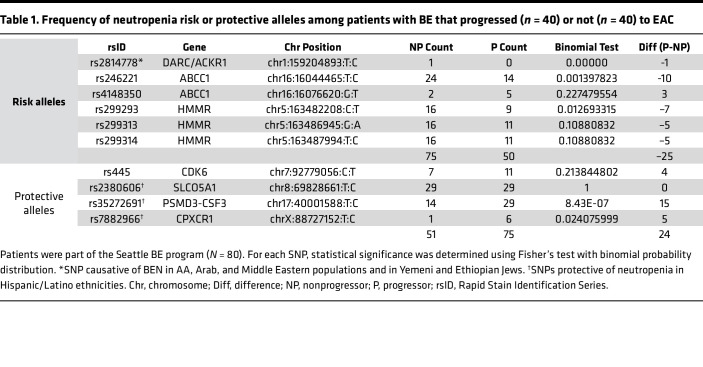
Frequency of neutropenia risk or protective alleles among patients with BE that progressed (*n =* 40) or not (*n =* 40) to EAC

## Version 1 09/22/2022

Electronic publication
