# Supplementary material for: AI-assisted discovery of an ethnicity-influenced driver of cell transformation in esophageal and gastroesophageal junction adenocarcinomas
Source: JCI Insight. 2022 Sep 22;7(18):e161334. doi: 10.1172/jci.insight.161334 (PMC9675486; doi:10.1172/jci.insight.161334)
Supplement: Supplemental data [file jciinsight-7-161334-s182.pdf]

AI-assisted Discovery of an Ethnicity-influenced Driver of Cell Transformation in Esophageal and Gastroesophageal Junction Adenocarcinomas

Authors: Pradipta Ghosh[1-4*†], Vinicius J. Campos[5*], Daniella T. Vo[6], Caitlin Guccione[7], Vanae Goheen-Holland[1, 3], Courtney Tindle[1, 3], Guilherme S. Mazzini[5, 8], Yudou He[1, 4], Ludmil Alexandrov[1, 4], Scott M. Lippman[2, 4], Richard R. Gurski[5,8,9], Soumita Das[3, 10], Rena Yadlapati[2*], Kit Curtius[2, 7*], and Debashis Sahoo[4, 6, 11*†]

Affiliations: [1]Department of Cellular and Molecular Medicine, University of California San Diego; [2] Department of Medicine, University of California San Diego; [3] HUMANOID Center of Research Excellence (CoRE), University of California San Diego; [4] Moore's Comprehensive Cancer Center, University of California San Diego; [5] Department of Gastrointestinal Surgery, Hospital de Clínicas de Porto Alegre, 2350 Ramiro Barcellos Street, Porto Alegre, RS, 90035-003; [6] Department of Pediatrics, University of California San Diego; [7] Division of Biomedical Informatics, University of California San Diego; [8] Postgraduate Program in Medicine; Surgical Sciences, Federal University of Rio Grande do Sul, 2400 Ramiro Barcellos Street, Porto Alegre, RS, 90035-003; [9] Medical School of Federal University of Rio Grande do Sul 2400 Ramiro Barcellos Street, Porto Alegre, RS, 90035-003; [10] Department of Pathology, University of California San Diego; [11] Department of Computer Science and Engineering, Jacob's School of Engineering, University of California San Diego.

[†]Equal contributions.

*CORRESPONDING AUTHOR CONTACT INFORMATION

Debashis Sahoo, Ph.D.; Associate Professor, Department of Pediatrics, University of California San Diego; 9500 Gilman Drive, MC 0703, Leichtag Building 132; La Jolla, CA 92093-0703. Phone: 858-246-1803: Fax: 858-246-0019: Email: dsahoo@ucsd.edu

Vinicius Campos, M.D.; Department of Gastrointestinal Surgery, Hospital de Clínicas de Porto Alegre, 2350 Ramiro Barcellos Street, Porto Alegre, RS, 90035-003. Email: viniciusjcampos@gmail.com

Rena Yadlapati, **M.D**.; Associate Professor, Division of Gastroenterology; Department of Medicine, University of California San Diego; 9500 Gilman Drive, MC 0703, La Jolla, CA 92093-0887. Email: ryadlapati@health.ucsd.edu

Kathleen Curtius, **Ph.D**.; Assistant Professor, Division of Biomedical Informatics, Department of Medicine, University of California San Diego; 9500 Gilman Drive, MC 0703, Biomedical Research Facility-2; La Jolla, CA 92093-0728. Phone: 619-777-3296: Email: kcurtius@health.ucsd.edu

Pradipta Ghosh, M.D.; Professor, Departments of Medicine and Cell and Molecular Medicine, University of California San Diego; 9500 Gilman Drive (MC 0651), George E. Palade Bldg, Rm 232; La Jolla, CA 92093. Phone: 858-822-7633: Email: prghosh@ucsd.edu

This PDF file includes:

- **Supplementary Text**
- **Detailed Materials and Methods**
- **Supplementary Figures. S1 to S10**
- **Supplementary Tables 6** (separately loaded as Supplemental Information excel sheets, with large datasets)

Supplementary Text

i. Creation of a Boolean map of metaplastic progression in the esophagus

We used Boolean Network Explorer (*BoNE*)[1] to first create a model of progressive gene regulatory events that occur during metaplastic transition (**Figure 2A**). For model training and development, we used the largest (to our knowledge) well annotated transcriptomic dataset [n = 76: GSE100843[2]] derived from BE and proximal matched normal mucosa from squamous esophagus from 18 BE patients. Gene expression patterns were first simplified into 'clusters' of genes equivalent to each other (**Figure 2A**-2). The clusters (nodes) were connected to one another based on the pattern of relationships between the clusters (edges), conforming to one of the six possible Boolean implication relationships (BIRs; **Figure 2A**-3). These efforts helped chart numerous Boolean paths (**Figure 2A**, 4-*left*) within a network with directed edges. Each cluster was then evaluated for whether they belong to the healthy esophagus or diseased side (BE) depending on whether the average gene expression value of a cluster in heathy samples is up or down, respectively. Each path of connected gene clusters indicates a certain hierarchy in gene expression events, which translates to a progressive series of gene down/upregulation events, predicted to occur in sequence during the metaplastic process (**Figure 2A**, 4-*right*).

We next introduced in *BoNE* machine learning that seeks to identify which of the gene clusters (nodes) connected by Boolean implication relationships (edges) are most optimal in distinguishing healthy from diseased samples. *BoNE* computes a score that naturally orders the samples; this score can be thought of as a continuum of states. A set of two clusters emerged as most robust, which was further refined by an additional filtering step through a second 'training dataset' [GSE39491[3]; see Supplemental Information 1] which is comprised of BE and normal esophageal matched samples from 43 patients. Both training datasets were analyzed independently throughout the process. The resultant model of metaplastic transition pinpointed a time series of metaplasia (BE)-associated invariant events, in which downregulation of expression of 220 genes (*SPINK7*-cluster; **Figure 2B-C**) was invariably associated with a concomitant upregulation of 24 genes (*SLC44A4*-cluster, **Figure 2B-C**) in all samples in the training datasets. *SPINK7* (serine peptidase inhibitor, kazal type 7), is a key checkpoint in the esophageal keratinocyte stem cell, which regulates mucosal differentiation, barrier function, and inflammatory responses[4]. *SLC44A4* encodes a specific high-affinity regulated carrier-mediated uptake system for TPP in human colonocytes, involved in regulation of microbiota-generated thiamine[5].

Reactome pathway analysis of the upregulated *SLC44A4*- and downregulated *SPINK7*-clusters along the path continuum revealed the most important biological processes that they control (**Figure S1**). The downregulated pathways (**Figure S1A**) were cellular processes that are inherently associated with squamous epithelium, e.g., keratinization, cornified envelope formation, as expected. Other notable changes were TP53 expression and cell-cell adhesion proteins. These findings are consistent with emerging evidence from numerous independent studies which agree that aberrant TP53 IHC highly correlated with *TP53* mutation status (90.6% agreement) and was strongly associated with higher risk of neoplastic progression regardless of the presence/absence of dysplasia[6-8]. The findings are also in keeping with the reduction observed by IHC in cell adhesion proteins in BE lesions [E-cadherin, P-cadherin and the catenins which serve as adaptor proteins that enable the cadherins to achieve cell adhesion[9]]. The most notable cellular processes that were upregulated (**Figure S1B**) were related to oxygen delivery to the tissue, consistent with reports of Warburg and Crabtree effects in BE tissues[10].

ii. Currently available animal models of BE→EAC transformation rarely recapitulate human disease

Animal models of diseases have both merits and limitations[11, 12]. Because 'mice are not men'[13, 14], especially when it comes to their innate immune system[14, 15], and EACs and GEJ-ACs are associated with a prominent immune signature, we asked how well currently available EAC models recapitulate the human disease (**Figure S5A**). To model how EAC-associated risk factors, i.e., obesity/BMI and IL8-induction enhance cell transformation, mice challenged with high-fat diet (HFD[16-18]) or overexpressing IL8[18] have been developed. Neither model induce our network-derived BE/EAC signatures (**Figure S5B**, *row i-iii*; **Figure S5D-E**); nor did they display induction of the neutrophil signatures we observed in human tissues (compare human-**Figure 4G**

with murine- **Figure S5C**, *row i-iii*). The signatures were, however, induced in a transgenic interleukin1-β (IL1β)-overexpression model (GSE24931; **Figure S5B-C**; *rows iv-vi;* **Figure S5F-H**); in this model, the human IL1β cDNA was inserted downstream of an Epstein-Barr virus (ED-L2) promoter that targets the oral cavity, esophagus, and squamous forestomach[19]. These mice develop chronic inflammation that progresses to BE and EAC; progression was accelerated by exposure to bile acids. Findings show that a combination of inflammation and bile acids, the latter are components of gastroduodenal reflux that has been linked to BE→EAC progression[19, 20]. Most importantly, the bile acid-accelerated model (GSE24931) recapitulated the neutrophil processes that were encountered in most human datasets of EACs and GEJ-ACs (**Figure 4F'**).

Materials and Methods

Computational Approaches

An AI-assisted study design that uses Boolean approach to build transcriptomic networks

We chose a Boolean approach to building transcriptomic networks[21] because of its ability to pinpoint with precision cellular states in tissues. For example, it helped pinpoint branchpoints in B/T cell differentiation[22, 23], define progenitor cell hierarchy in blood[24-28], normal and neoplastic cell states in colorectal cancers[29, 30], bladder cancers[31-34], and prostate cancers[35, 36], and identify NK cell exhaustive states[37], universal cell proliferative[38] and macrophage[39] markers, and cell states in the mucosal barrier in IBD[40]. Because the Boolean approach relies on invariant relationships that are conserved despite heterogeneity in the samples used for the analysis, which often represent maximum possible diversity, i.e., the relationships can be thought of as general relationships among pairs of genes across all samples irrespective of their origin (normal or disease), laboratories/cohorts, different perturbations, and sometimes in multiple species including human, mouse and rat, and hence, considered conserved invariants. It is assumed that such 'invariants' are likely to be fundamentally important for any given process.

Barrett's and Esophageal Adenocarcinoma datasets used for network analysis

One microarray dataset (GSE100843; n = 76, 36 Normal esophageal squamous mucosa and 40 Barrett's esophagus segment) is used to perform Boolean Implication network analysis of Barrett's esophagus (BE) samples, and another microarray dataset (GSE39491, n = 80, 40 normal, 40 BE) is used to train a network model to distinguish normal vs BE samples. Boolean Implication Network analysis of the Esophageal Adenocarcinoma (EAC) is performed using RNA-seq dataset (E-MTAB-4054, n = 63, 19 normal, 19 BE without dysplasia, 8 BE with low-grade dysplasia, 17 EAC). All training and validation dataset (**Supplementary Information 1**) were downloaded from National Center for Biotechnology Information (NCBI) Gene Expression Omnibus website (GEO)[41-43] or European Molecular Biology Laboratory (EMBL) European Bioinformatics Institute (EMBL-EBI) ArrayExpress website[44]. All gene expression datasets (**Supplementary Information 1**) were processed separately using the Hegemon data analysis framework [29, 30, 32]. We did not combine datasets that belong to two different platforms. See **Supplemental Information 1** for the degree of heterogeneity among samples in the datasets used in this work.

Test cohort selection

Two different test cohorts were used to build the network and perform machine learning for BE: GSE100843 and GSE39491. Both GSE100843 and GSE39491 are microarray datasets that included reasonable number (>= 30) of normal esophageal squamous mucosa and reasonable number (>= 40) of BE samples. Since the number of samples in these cohort are less than 100, which is on the lower side for comprehensive Boolean analysis, network is built on GSE100843 (n = 76) and machine learning is performed on an independent dataset GSE39491 (n = 80) to cover the entire spectrum of gene expression dataset from different microarray platforms. Surprisingly, variation in gene expression were good enough to use our standard BooleanNet statistic ($S > 3$ and $p < 0.1$) to identify Boolean Implication relationships with n = 76. Only one cohort was used to build the network and perform machine learning for EAC: E-MTAB-4054. E-MTAB-4054 (n = 63) is the only large RNASeq dataset available that provided high-quality measurements of mRNA extracted from normal, BE and EAC tissue samples. Since all these cohorts have small number of samples, reliability of Boolean analysis is low, and the results need to be supported by large and strong groups of validation datasets.

Boolean Analysis

Boolean logic is a simple mathematic relationship of two values, i.e., high/low, 1/0, or positive/negative. The Boolean analysis of gene expression data requires first the conversion of expression levels into two possible values. The *StepMiner* algorithm is reused to perform Boolean analysis of gene expression data [21]. The *Boolean analysis* is a statistical approach which creates binary logical inferences that explain the relationships between phenomena. Boolean analysis is performed to determine the relationship between the expression levels of pairs of genes. The *StepMiner* algorithm is applied to gene expression levels to convert them into Boolean values (high and low). In this algorithm, first the expression values are sorted from low to high and a rising step function is fitted to the series to identify the threshold. Middle of the step is used as the *StepMiner* threshold. This threshold is used to convert gene expression values into Boolean values. A noise margin of 2-fold change is applied around the threshold to determine intermediate values, and these values are ignored during Boolean

analysis. In a scatter plot, there are four possible quadrants based on Boolean values: (low, low), (low, high), (high, low), (high, high).

Invariant Boolean implication relationships

A Boolean implication relationship is observed if any one of the four possible quadrants or two diagonally opposite quadrants are sparsely populated. Based on this rule, there are six different kinds of Boolean implication relationships. Two of them are symmetric: equivalent (corresponding to the highly positively correlated genes), opposite (corresponding to the highly negatively correlated genes). Four of the Boolean relationships are asymmetric, and each corresponds to one sparse quadrant: (low => low), (high => low), (low => high), (high => high). BooleanNet statistics (Equations listed below) is used to assess the sparsity of a quadrant and the significance of the Boolean implication relationships [21, 22]. Given a pair of genes A and B, four quadrants are identified by using the StepMiner thresholds on A and B by ignoring the Intermediate values defined by the noise margin of 2-fold change (+/- 0.5 around StepMiner threshold). Number of samples in each quadrant are defined as a_{00}, a_{01}, a_{10}, and a_{11}. Total number of samples where gene expression values for A and B are low is computed using following equations.

$$nA_{low} = (a_{00} + a_{01}), nB_{low} = (a_{00} + a_{10}),$$

Total number of samples considered is computed using following equation.

$$total = a_{00} + a_{01} + a_{10} + a_{11}$$

Expected number of samples in each quadrant is computed by assuming independence between A and B. For example, expected number of samples in the bottom left quadrant $e_{00} = \hat{n}$ is computed as probability of A low ((a_{00} + a_{01})/total) multiplied by probability of B low ((a_{00} + a_{10})/total) multiplied by total number of samples. Following equation is used to compute the expected number of samples.

$$n = a_{ij}, \hat{n} = (nA_{low}/total * nB_{low}/total) * total$$

To check whether a quadrant is sparse, a statistical test for ($e_{00} > a_{00}$) or ($\hat{n} > n$) is performed by computing S_{00} and p_{00} using following equations. A quadrant is considered sparse if S_{00} is high ($\hat{n} > n$) and p_{00} is small.

$$S_{ij} = \frac{\hat{n} - n}{\sqrt{\hat{n}}}$$

$$p_{00} = \frac{1}{2}\left(\frac{a_{00}}{(a_{00} + a_{01})} + \frac{a_{00}}{(a_{00} + a_{10})}\right)$$

A threshold of S_{00} > sthr and p_{00} < pthr to check sparse quadrant. A Boolean implication relationship is identified when a sparse quadrant is discovered using following equation.

Boolean Implication = (S_{ij} > sthr, p_{ij} < pthr)

A relationship is called Boolean equivalent if top-left and bottom-right quadrants are sparse.

Equivalent = $(S_{01} > sthr,\ P_{01} < pthr, S_{10} > sthr, P_{10} < pthr)$

Boolean opposite relationships have sparse top-right (a_{11}) and bottom-left (a_{00}) quadrants.

$$Opposite = (S_{00} > sthr,\ P_{00} < pthr, S_{11} > sthr, P_{11} < pthr)$$

Boolean equivalent and opposite are symmetric relationship because the relationship from A to B is same as from B to A. Asymmetric relationship forms when there is only one quadrant sparse (A low => B low: top-left; A low => B high: bottom-left; A high=> B high: bottom-right; A high => B low: top-right). These relationships are asymmetric because the relationship from A to B is different from B to A. For example, A low => B low and B low => A low are two different relationships.

A low => B high is discovered if bottom-left (a_{00}) quadrant is sparse and this relationship satisfies following conditions.

A low => B high = $(S_{00} > sthr,\ P_{00} < pthr)$

Similarly, A low => B low is identified if top-left (a_{01}) quadrant is sparse.

A low => B low = $(S_{01} > sthr,\ P_{01} < pthr)$

A high => B high Boolean implication is established if bottom-right (a_{10}) quadrant is sparse as described below.

A high => B high = $(S_{10} > sthr,\ P_{10} < pthr)$

Boolean implication A high => B low is found if top-right (a_{11}) quadrant is sparse using following equation.

A high => B low = $(S_{11} > sthr,\ P_{11} < pthr)$

For each quadrant, a statistic S_{ij} and an error rate p_{ij} is computed. $S_{ij} > 3$ and $p_{ij} < 0.1$ are the thresholds used on the BooleanNet statistics to identify Boolean implication relationships (BIRs). False discovery rate is computed by randomly shuffling each gene and computing the ratio of the number of Boolean implication relationship discovered in the randomized dataset and original dataset. The false discovery rate for BE and EAC dataset was less than 0.001.

Boolean Implication analysis looks for invariant relationship across all the different types of samples regardless of the conditions and treatment protocols. Therefore, it does not distinguish the sample types when discovering Boolean implication relationships. We assume that there are fundamental invariant Boolean implication formula that are satisfied by every sample regardless of their type.

Construction of BE/EAC Boolean Implication Networks

A Boolean implication network (BIN) is created by identifying all significant pairwise Boolean implication relationships (BIRs)[44, 45]. The Boolean implication network contains the six possible Boolean relationships between genes in the form of a directed graph with nodes as genes and edges as the Boolean relationship between the genes. The nodes in the BIN are genes and the edges correspond to BIRs. Equivalent and Opposite relationships are denoted by undirected edges and the other four types (low => low; high => low; low => high; high => high) of BIRs are denoted by having a directed edge between them. The network of equivalences seems to follow a scale-free trend; however, other asymmetric relations in the network do not follow scale-free properties. BIR is strong and robust when the sample sizes are usually more than 200 (from our experience of using Boolean Implication for more than 10 years). All our previous papers use thousands of diverse samples to establish Boolean implication relationships. However, Boolean Implication analysis is carried out in such low number of samples such as the selected GSE100843 (n =76) and E-MTAB-4054 (n = 63) datasets. We have demonstrated that we have a reasonable False Discovery Rate (< 0.001) when S > 3 and p < 0.1 are used. Both GSE100843 and E-MTAB-4054 dataset were prepared for Boolean analysis by filtering genes that had a reasonable dynamic range of expression values. When the dynamic range of expression values was small, it was difficult to distinguish if the values were all low or all high or there were some high and some low values. Thus, it was determined to be best to ignore them during Boolean analysis. The filtering step was performed by analyzing the fraction of high and low values identified by the StepMiner algorithm [46]. Any probe set or genes which contained less than 5% of high or low values were dropped from the analysis.

Generation of Clustered Boolean Implication network

Clustering was performed in the Boolean implication network to dramatically reduce the complexity of the network. A clustered Boolean implication network (CBIN) was created by clustering nodes in the original BIN by following the equivalent BIRs. One approach is to build connected components in an undirected graph of Boolean equivalences. However, because of noise, the connected components become internally inconsistent e.g., two genes opposite to each other become part of the same connected component. In addition, the size of clusters became unusually big with almost everything in one cluster. To avoid such a situation, we need to break the component by removing the weak links. To identify the weakest links, we first computed a minimum spanning tree for the graph and computed the Jaccard similarity coefficient for every edge in this tree. Ideally if two members are part of the same cluster, they should share as many connections as possible. A threshold is considered for the Jaccard similarity coefficient (0.8 for BE network and 0.5 for the EAC network) below which the edges are dropped from further analysis. Thus, many weak equivalences were dropped using the above algorithm leaving the clusters internally consistent. We removed all edges that have Jaccard similarity coefficient less than the selected threshold and built the connected components with the rest. The connected components were used to cluster the BIN which is converted to the nodes of the CBIN. The choice of the threshold on the Jaccard similarity coefficient play an important role in determining the size and the number of clusters as well as whether they are internally consistent. A new graph was built that connected the individual clusters to each other using Boolean relationships. The link between two clusters (A, B) was

established by using the top representative node from A that was connected to most of the members of A and sampling 6 nodes from cluster B and identifying the overwhelming majority of BIRs between the nodes from each cluster.

A CBIN was created using the GSE100843 and E-MTAB-4054 datasets. The edges between the clusters represented the Boolean relationships that are color-coded as follows: orange for low => high, dark blue for low => low, green for high => high, red for high => low, light blue for the equivalent and black for the opposite. A subnetwork is selected using low=>low (blue), high => low (red) and opposite (merged with high=>low as red) edges among the top 10 clusters.

Charting Boolean paths

Boolean paths have been explored before to predict the underlying time series events in biological processes such as B cell differentiation [22, 23] and early differentiation events in cancer stem cell [29, 30, 32, 35]. This algorithm is called MiDReG (Mining Developmentally Regulated Genes) that uses two seed genes to identify intermediate genes in a biological process. MiDReG infer intermediate states using a sequence of asymmetric BIRs. Here, using MiDReg algorithm/concept to traverse the Boolean Implication network that identifies paths of clusters where the start and end clusters in the clustered Boolean implication network mark the end points of a possible set of events from healthy to disease. The asymmetric BIRs provide a unique dimension to the network that is fundamentally different from any other gene expression networks in the literature. Traversing a set of nodes in a directed graph of the Boolean network constitutes a Boolean path. A simple Boolean path involves two nodes and the directed edge between them. A complex Boolean path involves more than two nodes and the edges between them.

Ordering samples based on composite score of Boolean path

A Boolean path contains one or more clusters. A composite score is computed for each cluster and combined later. To compute the final score, first the genes present in each cluster were normalized and averaged. Gene expression values were normalized according to a modified Z-score approach centered around StepMiner threshold (formula = (expr - SThr)/3/stddev). A weighted linear combination of the averages from the clusters of a Boolean path was used to create a score for each sample. The weights along the path either monotonically increased or decreased to make the sample order consistent with the logical order based on BIR. The samples were ordered based on the final weighted and linearly combined score. A cluster highly expressed in a disease setting received a weight of 1 and healthy setting received a weight of -1.

Summary of genes in the clusters

Reactome pathway analysis of each cluster along the top continuum paths was performed to identify the enriched pathways[47]. The pathway description was used to summarize at a high-level what kind of biological processes are enriched in a particular cluster. These can be accessed in **Supplementary Information 2** (for BE) and **Supplementary Information 3** (for EAC).

Measurement of classification strength or prediction accuracy

Receiver operating characteristic (ROC) curves were computed by simulating a score based on the ordering of samples that illustrates the diagnostic ability of binary classifier system as its discrimination threshold is varied along with the sample order. The ROC curves were created by plotting the true positive rate (TPR) against the false positive rate (FPR) at various threshold settings. The area under the curve (often referred to as simply the AUC) is equal to the probability that a classifier will rank a randomly chosen IBD samples higher than a randomly chosen healthy samples. In addition to ROC AUC, other classification metrics such as accuracy ((TP + TN)/N; TP: True Positive; TN: True Negative; N: Total Number), precision (TP/(TP+FP); FP: False Positive), recall (TP/(TP+FN); FN: False Negative) and f1 (2 * (precision * recall)/(precision + recall)) scores were computed. Precision score represents how many selected items are relevant and recall score represents how many relevant items are selected. Fisher exact test is used to examine the significance of the association (contingency) between two different classification systems (one of them can be ground truth as a reference).

AI guided discovery of Boolean paths

A Boolean path is converted to a path score as mentioned above using a linear combination of normalized gene expression values. The strength of classification of normal and BE/EAC samples using this score is computed by the ROC-AUC measurement. We ranked the clusters based on the ROC-AUC values in the cohort (GSE39491, n = 80, 40 normal, 40 BE) for BE network and E-MTAB-4054 (27 BE vs 17 EAC). Multivariate regression is performed to select the best clusters. The clusters for BE network are filtered by enrichment of differentially expression genes from a recently published BE model (GSE153129). Only two clusters (*SPINK7* and *SLC44A4*) are selected based on this filter: SPINK7 cluster is down-regulated in BE and SLC44A4 cluster is up-regulated in BE.

Training and Validation Datasets

A Boolean path is selected after machine learning to construct a Boolean model. The Boolean model is tested in several human and mouse datasets, each comprised of a heterogeneous collection of samples (as mentioned in **Supplementary Information 1**) to demonstrate reproducibility. Selected Boolean path score is computed as mentioned in section "*Ordering samples based on composite score of Boolean path*". The sample order using the Boolean path score is evaluated using the sample annotation (normal vs BE; BE vs EAC) by ROC-AUC analysis. We tested how the SPINK7-SLC44A4 path score with weight -1, 1 respectively distinguishes normal and BE samples as they are annotated in training datasets (GSE100843, GSE39491), and validation datasets (GSE65013, GSE64894, GSE49292, GSE26886, GSE34619, GSE13083, E-MTAB-4054). Top two EAC network clusters are selected based on the best ROC-AUC values using the training cohort (E-MTAB-4054): IL10RA, LILRB3. Both these clusters are upregulated in EAC compared to BE samples. The IL10RA-LILRB3 path score is tested with weight 1, 1 respectively to distinguish BE vs EAC samples in training dataset (E-MTAB-4054) and validation datasets (GSE26886, GSE37200, GSE77563). The EAC score is also tested to distinguish normal vs EAC+GEJAC in GSE74553 cohort. We have collected publicly available gene expression datasets derived from mouse models of BE/EAC (GSE24931, GSE103616, GSE158116; **Supplementary Data 1**) to test whether human Boolean models perform well in mice. The gene name conversion from human to the mouse is performed using human genome GRCh38.95 ensembl IDs and mapping data exported from ensemble BioMart web-interface.

Mathematical model of NE-BE-EAC Progression

MiDReG[22] (Mining Developmentally Regulated Genes) algorithm is used to model disease progression using concept of invariant Boolean implication formula. A Boolean implication formula is considered invariant if the formula is consistent in almost all samples from a particular domain (almost no exceptions). We observed a Boolean implication formula *SPINK7* high => *SLC44A4* low, that is consistent in most diverse global human GSE119087 (n = 25955) dataset. We assumed that this invariant will also hold in NE, BE and EAC samples. When we focus only on the NE, BE and EAC samples, we observed the Boolean implication *CXCL8* high => *SLC44A4* high (S > 2, p < 0.1; FDR < 0.001) in multiple independent cohorts [E-MTAB-4054, and Pooled GSE26886 (n=58) + GSE40220 (n=3) + GSE42363 (n=14)]. However, combined NE (n=20), BE (n = 20), EAC (n=35), and ESCC (n = 407) samples pooled from GSE26886 (n=67), GSE40220 (n=3), GSE42363 (n=14), GSE69925 (n=266), GSE77861 (n=7), GSE161533 (n=28), GSE32701 (n=29), GSE106185 (n=23), GSE45670 (n=28), GSE17351 (n=5), GSE44021 (n=6), GSE100942 (n=4) and GSE33810 (n=2) failed to show Boolean implication CXCL8 high => SLC44A4 high (S = -0.76, p = 0.91). ESCC samples populate in the CXCL8 high and SLC44A4 low quadrant. All of the pooled samples were microarray datasets generated using the [HG-U133_Plus_2] Affymetrix Human Genome U133 Plus 2.0 Array GPL570 platform. The pooled samples are re-normalized together using RMA algorithm[48]. The NE, BE, and EAC samples nicely organize themselves in three different quadrants (*CXCL8* low and *SLC44A4* low, *CXCL8* low and *SLC44A4* high, *CXCL8* high and *SLC44A4* high) respectively. Matched normal or adjacent non-tumor samples were excluded from the pooled datasets because of contamination from tumor samples (many of them have high levels of *CXCL8* expression patterns). Only two normal samples behaved as outliers removed from the analysis (GSM661768 behaved like BE and GSM661777 behaved like ESCC samples). Mathematical model of invariant makes it clear that BE must precede EAC during disease progression.

<u>Outcome studies</u>:

Patient cohort

We retrospectively analyzed a previously assembled dataset[49] of patients with biopsies reporting BE between 2013 and 2017 and with a complete blood count within 6 months from the endoscopy, as well as patients with esophageal

adenocarcinoma (EAC). Cases (n = 113) were classified as non-dysplastic BE (NDBE, n = 72), dysplastic BE (DBE, n = 11) and EAC (n = 30).

Briefly, to enroll the patients with BE, medical records of all patients undergoing upper endoscopy at a tertiary care center in Brazil (Hospital de Clínicas dePorto Alegre) between January 2013 and September 2017 who had columnar epithelium visualized in the distal esophagus were retrospectively analyzed. All endoscopic examinations that fulfilled criteria were included, even though belonging to the same patient in a different period of the surveillance. Exclusion criteria were absence of confirmed intestinal metaplasia on histology (goblet cells on Alcian-Blue staining), immunosuppression by drugs or chronic diseases, active or recent (< 6 months) infectious disease, history of cancer or any hematological or autoimmune disease, and previous surgery of the gastrointestinal tract (except fundoplication for GERD). To be considered eligible, cases also must have had a complete blood count (CBC) collected between the period of 6 months before and 6 months after endoscopy and necessarily outside of a context of clinical emergency (for example, on the emergency room, for any reason) or invasive procedures (such as surgery or esophageal dilation). Those criteria were assessed through a thorough exam of electronic medical records. In the presence of more than one eligible CBC, the one closest to the day of the endoscopy was selected. If an included patient had any endoscopy performed before 2013, those exams were also analyzed for inclusion according to the criteria stated above.

Patients with EAC were selected from hospital discharge diagnostic records between January 2005 and December 2017 if they had the following codes, according to the international classification of diseases (ICD-10; C15: malignant neoplasm of the esophagus): C15.2 (abdominal esophagus), C15.5 (lower third), C15.8 (overlapping sites), and C15.9 (unspecified). Squamous cell carcinoma and gastroesophageal junction (GEJ) tumor types 2 or 3 of Siewert classification14 were excluded. Same exclusion criteria used for BE cases were applied, except the need of confirmed intestinal metaplasia.

Demographic, clinical, and endoscopic data collected (as published before[49]). Data collected from endoscopic histopathological reports were histologic diagnosis and adherence to Seattle Protocol, that was evaluated comparing endoscopic descriptions with pathology reports (adherence was considered if sent to pathology separated samples with at least 4 biopsy fragments for every 2 cm of columnar epithelia). Histopathological diagnosis of intestinal metaplasia, dysplasia, and adenocarcinoma was carried out by expert pathologists in the institution in a clinical routine fashion.

All cases were classified into three groups based on histopathological diagnosis: non-dysplastic BE (NDBE), dysplastic BE (DBE)—with either low- or high-grade dysplasia—and esophageal adenocarcinoma (EAC). Staging of EAC patients was done based on TNM classification according to the 7th edition of American Joint Committee on Cancer (2010). EAC patients were divided in 2 groups, according to TNM (stage I/II and III/IV). For patients submitted to esophagectomy without neoadjuvant treatment, pathological stage was used; clinical staging was considered for the rest.

The study was approved by the Brazilian National Committee on Research Ethics (CONEP), registered by number CAAE-81068617.2.0000.5327.

Univariate and multivariate analyses

Prediction of progression from NDBE to DBE to EAC is analyzed using univariate and multivariate analyses based on several clinical parameters such as SEX, ALC, ANC, and PLAT. Univariate and multivariate analyses is performed using Ordinary Least Squares regression (OLS) python statsmodels (version 0.12.2) package.

Survival analysis in TCGA-EASC and TCGA-STAD dataset

TCGA-EASC and TCGA-STAD datasets were used to study the relationship between the composite expression score of different genes (or single CXCL8 gene) from various signatures and patient clinical outcomes for esophageal adenocarcinoma (EAC), esophageal squamous cell carcinomas (ESCC) and gastric adenocarcinomas (GC). We evaluated the prognostic value of mRNA expression of either single (CXCL8) or several gene combinations according to overall survival (OS) in cancer patients with high mutation load. Nonsynonymous mutations were discovered using 'missense-variant', 'splice-region-variant', 'splice-donor-variant', 'frameshift-variant', 'stop-gained', 'inframe-deletion', 'splice-acceptor-variant', 'coding-sequence-variant', 'non-coding-transcript-exon-variant', 'non-coding-transcript-variant', 'inframe-insertion', 'start-lost', 'stop-lost', 'NMD-transcript-variant', 'protein-altering-variant' and 'incomplete-terminal-codon-variant' annotations. High mutation load is computed using median threshold of the number of mutations observed in tumor samples based on four different mutation callers from the TCGA portal summarized in UCSC Xena browser: VarScan, MuSE, MuTect, and SomaticSniper. A total of 65 patients with EAC, 26 patients with ESCC and 170 patients with GC were included in all analyses and events were plotted for a 4-year period (1460 days). StepMiner tool was used to compare the predictive value

of several gene combinations in patients with low and high expressions. Patients were divided into two groups, high vs. low expression, based on the StepMiner threshold +/- noise margin of gene expression or the composite expression score. Noise margin of a composite signature is estimated by the square root of the sum of squares of the scaled down (1/3/stddev) version of the individual noise margin of the gene which is 0.5 based on the BooleanNet approach. The OS Kaplan-Meier plots are presented with the log-rank *p*-value. For the composite signature scores of CD16 (FCGR3A and FCGR3B) in EAC, threshold was computed by using StepMiner twice, first on the whole scores and then using all the values lower than the first StepMiner threshold. For ESCC, threshold for composite score of CD16 (FCGR3A and FCGR3B) was computed by using StepMiner twice, first on the whole scores and then using all the values greater than the first StepMiner threshold.

Genomic analyses:

Patient samples

Whole-genome sequencing data for 320 esophageal biopsies from 80 patients with Barrett's esophagus (BE) were analyzed (de-identified data publicly available, dbGaP Study Accession**:** phs001912 and phs001654). Longitudinal data from patients were collected as part of a case-control study design performed at the Fred Hutchinson Cancer Research Center. Demographics for patient cohort have been previously described[50]. Progressors (cases) were defined as patients who progressed to esophageal adenocarcinoma (EAC) during surveillance (n=40 total) in the Seattle Barrett's Esophagus Program. Non-progressors (controls) were defined as those who did not progress to EAC throughout long-term follow-up (n=40 total).

Mutational analysis

After downloading the BE genomics data, quality control checks were performed for each sample to remove any sequencing files that do not meet our stringent criteria. Briefly, samples were greylisted when: (i) FASTQ file(s) failed one or more of the criteria from FastQC1; (ii) a tumor-normal pair had less than 95% concordance as estimated by Conpair2; (iii) samples exhibited low tumor purity, as estimated by ascatNgs3; (iv) samples exhibited low sequencing coverage for either the cancer or the normal tissue. Additionally, after mutation calling was completed, any sample with more than 10% of mutations attributed to signatures of known sequencing artifacts was greylisted. Only two of the original BE samples failed these quality control checks. Note that while somatic mutations and germline variants were identified in all greylisted samples, these samples were not used for any subsequent analyses. Somatic mutations and germline variants were identified using our ensemble variant calling pipeline. Specifically, FASTQ files were aligned to human genome build GRCh38.d1.vd1 using BWA-MEM5. Duplicate reads were marked using Picard MarkDuplicate6. The alignment was further refined through local indel realignment and base score recalibration using the GATK toolkit7,8. Next, the paired cancer and normal BAM files were subjected to somatic mutation calling analysis using four state-of-the-art computational tools. More specifically, single base substitutions (SBSs) and small insertions and deletions (indels) were identified independently using Mutect29, VarScan210, Strelka211, and MuSE12. Germline single-nucleotide polymorphisms (SNPs) were detected using VarScan2. Copy-number alterations were identified using ascatNgs3. Somatic mutations marked as "PASS" by Mutect2 and Strelka2 were filtered based on their mutation confidence scores: TLOD score >= 10 (Mutect2) and SomaticEVS >= 15 (Strelka2). Any somatic mutations found in 2 or more of the 4 variant callers were considered as high confidence mutations; this optimum combination strategy is based on the previous experience from pan-cancer analysis performed by TCGA MC313 and ICGC PCAWG14. Subsequent filters were used to produce the final set of mutations for downstream analysis. The following filters addressed most artifact mutations introduced during sample preparation and/or sequencing: (i) DKFZbiasFiter15 removed SBSs with biased variant read support, which can be a result of unbalanced PCR amplification, biased reading during sequencing, and DNA shearing leading to 8-oxoguanine lesions during sample preparation16; (ii) panel of normal (PON) filter, generated using Mutect2, removed additional technical artifacts recurrently appearing in genomic locations of normal samples; (iii) variant allele frequency threshold was applied to all SBSs and indels to remove any low frequency mutations, which were commonly introduced as part of the DNA sequencing process17. Overall, this analysis generated a catalog of harmonized bona fide DNA somatic mutations and germline variants in each BE sample.

For downstream analyses, we further filtered for somatic mutations from this catalog that were found by all 4 callers described above. For each patient, we then considered the set of unique somatic mutations across their 4 samples from 2 timepoints (including 2 sampling locations of the upper esophagus and lower esophagus regions at each timepoint).

Statistical analysis

To calculate the number of occurrences of mutations in the cluster of genes identified from the BE→EAC map (**Figure S8A**), we cycled through merged VCF files from EAC Progressors (n=40) and summed the total number of mutations in each patient across the genes in each cluster as shown in blue boxplots. The same calculations were performed for non-progressor patients (n=40) and total mutations are shown in red boxplots. P-values from two-sided Mann–Whitney *U*-tests are reported above each plot comparing Progressor and Non-progressor patients for each cluster (statistical tests performed using scipy.stats.mannwhitneyu in Python v3.9.7).

To calculate the number of occurrences of mutations in neutrophil function associated genes, we cycled through the merged VCF files from patients who were EAC Progressors (n=40) and summed the total count for each gene. These totals by gene were then compared to totals counted in the same way within the non-progressor patients (n=40), see **Figure S8B**.

Experimental Approaches:

Reagents and antibodies

Antibodies that were used in this work include rabbit serum anti-SUPT6H (ThermoFisher, #A300-801A) and rabbit serum anti-P63/TP63 (BosterBio, #PA2056). Details regarding these antibodies is provided below. ImmPRESS HRP Horse Anti-Rabbit IgG Polymer Detection Kit Peroxidase was purchased through Vector Laboratories (#MP-7401-50), and the 2-Component DAB pack was purchased from BioGenex (#HK542-XAKE), as was the hematoxylin (#HK100-9K).

Name	Dilution factor	Validation (method)	Validation document	Manufacturer	Catalog number
Polyclonal Anti-TP63 Antibody	1:500	Validated commercially (Tested in Flow Cytometry, IF, IHC, ICC, WB applications.)	https://www.bosterbio.com/anti-p63-antibody-pa2056-boster.html	Boster Biological	PA2056
SUPT6 Polyclonal Antibody	1:250	Validated commercially (Tested in IHC, IP, WB applications)	https://www.thermofisher.com/antibody/product/SUPT6H-Antibody-Polyclonal/A300-801A	Bethyl Laboratories	A300-801A

Human subjects with or without BE

Esophageal biopsies used for IHC were obtained from patients undergoing endoscopies as a part of their routine care and follow-up at UC San Diego's Center for Esophageal Diseases. Patients were recruited and consented using a study proposal approved by the Institutional Review Board of the University of California, San Diego Center for Esophageal Diseases GEODE (Gastro-Esophageal Oncogenesis, Dysmotility, & Evolution) Research Program, Division of Gastroenterology, University of California San Diego, following the protocol approved by the Human Research Protection Program (HRPP) Institutional Review Board (Project ID#200047). Mucosal biopsies were obtained during sedated upper gastrointestinal endoscopy using cold forceps from two cohorts: 1) Asymptomatic healthy, 2) BE. For all cohorts, biopsies were obtained from the squamous epithelium 5 cm proximal to the Z line. Additionally, for the BE cohort the BE segment was closely inspected under high-definition white light and narrow band imaging, and biopsies were obtained from the esophagus 2-5 cm proximal to the BE segment. The clinical phenotype and information were curated based on histopathology reports from Clinical Pathology and Chart check. For all the deidentified human subjects the information including age, ethnicity, gender, previous history of disease and medication were collected from the chart following the security and privacy rules outlined in the HIPAA (Health Insurance Portability and Accountability Act of 1996) legislation. Written informed consent was obtained from all participants. The study design and the use of human study participants was conducted in accordance with the criteria set by the Declaration of Helsinki.

Immunohistochemistry of patient esophageal samples

Formalin-fixed, paraffin-embedded (FFPE) tissue sections of 4 μm thickness were cut and placed on glass slides coated with poly-L-lysine, followed by deparaffinization and hydration. Heat-induced epitope retrieval was performed using tris EDTA (pH 9.0) in a pressure cooker using a rolling boil for 15 minutes. Tissue sections were incubated with 3% hydrogen peroxidase for 5 minutes to block endogenous peroxidase activity, followed by a one-hour incubation with 2.5% horse serum. Slides were then incubated with primary antibodies for 1.5 hours in a humidified chamber at room temperature. After primary incubation, slides were incubated with secondary antibodies (horse, anti-rabbit) for 30 minutes at room temperature and washed. Antibodies used for immunostaining; SUPT6H [1:250], TP63 [1:500]. Immunostaining was visualized using 3,3′-diaminobenzidine chromogen and counterstained with Mayer's Hematoxylin. Samples were quantitatively analyzed and scored based on the presence (positive) or absence (negative) of staining.

IHC Quantification

IHC images were randomly sampled at different 300x300 pixel regions of interest (ROI). The ROIs were analyzed using IHC Profiler[51]. IHC Profiler uses a spectral deconvolution method of DAB/hematoxylin color spectra by using optimized optical density vectors of the color deconvolution plugin for proper separation of the DAB color spectra. The histogram of the DAB intensity was divided into 4 zones: high positive (0 to 60), positive (61 to 120), low positive (121 to 180) and negative (181 to 235). High positive, positive, and low positive percentages were combined to compute the final percentage positive for each region of interest (ROI). The range of values for the percent positive is compared among different experimental groups. Data is displayed as percent positive stain.

Statistical analysis

Statistical significance between experimental groups was determined using two-tailed Mann-Whitney test (IHC score). For all tests, a p-value of 0.05 was used as the cutoff to determine significance. All experiments were repeated a least three times, and p-values are indicated in each figure. All statistical analysis was performed using GraphPad prism 8.4.3.

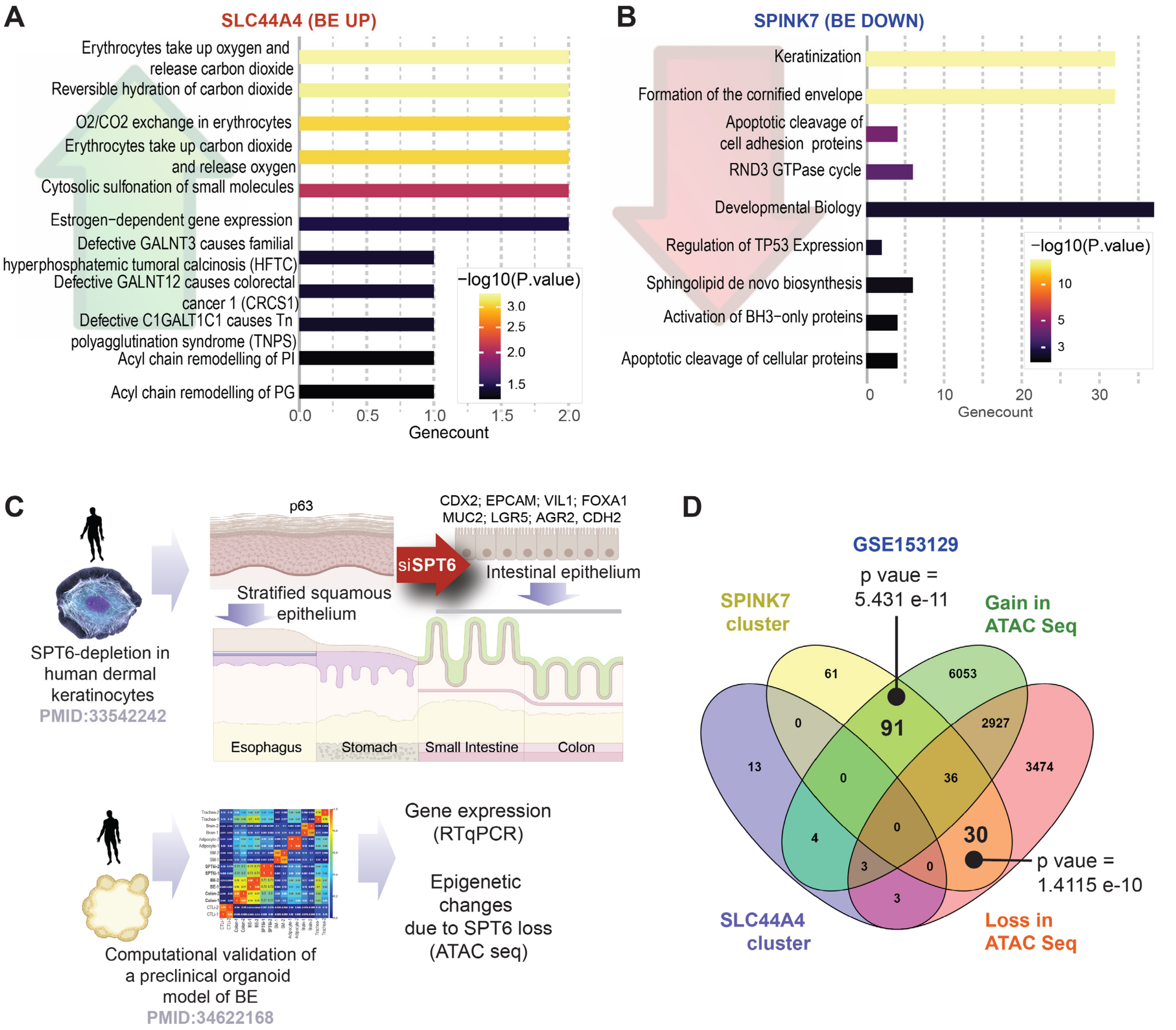

Figure S1. Generation and validation of Boolean network map of Barrett's metaplasia of the esophagus. [Related to Figure 2]

A-B. Reactome pathway (www.reactome.org) analysis of *SLC44A4*- (downregulated genes; A) and *SPINK7*- (upregulated genes; B) clusters were performed to identify the signaling pathways and cellular processes that are enriched during metaplastic progression in esophagus.

C. Schematic summarizing the key findings in gene expression and epithelial morphology observed and reported earlier [52], upon depletion of SPT6 in keratinocyte stem cells by siRNA [52]. While control keratinocytes formed stratified squamous epithelium, siRNA mediated transient depletion of SPT6 in keratinocytes (SPT6i) grew as 'intestine-like' monolayers. RNA seq studies on those monolayers confirmed that this model recapitulates metaplastic gene signatures in BE, and not normal gut differentiation that is observed in the healthy gut lining.

D. Gene clusters from the BE-map were analyzed for overlap with those affected in the organoid BE model by loss or gain of access due to chromatin remodeling upon SPT6 loss, as identified by ATAC Seq. Only significant *p values*, as determined using hypergeometric analyses, are displayed.

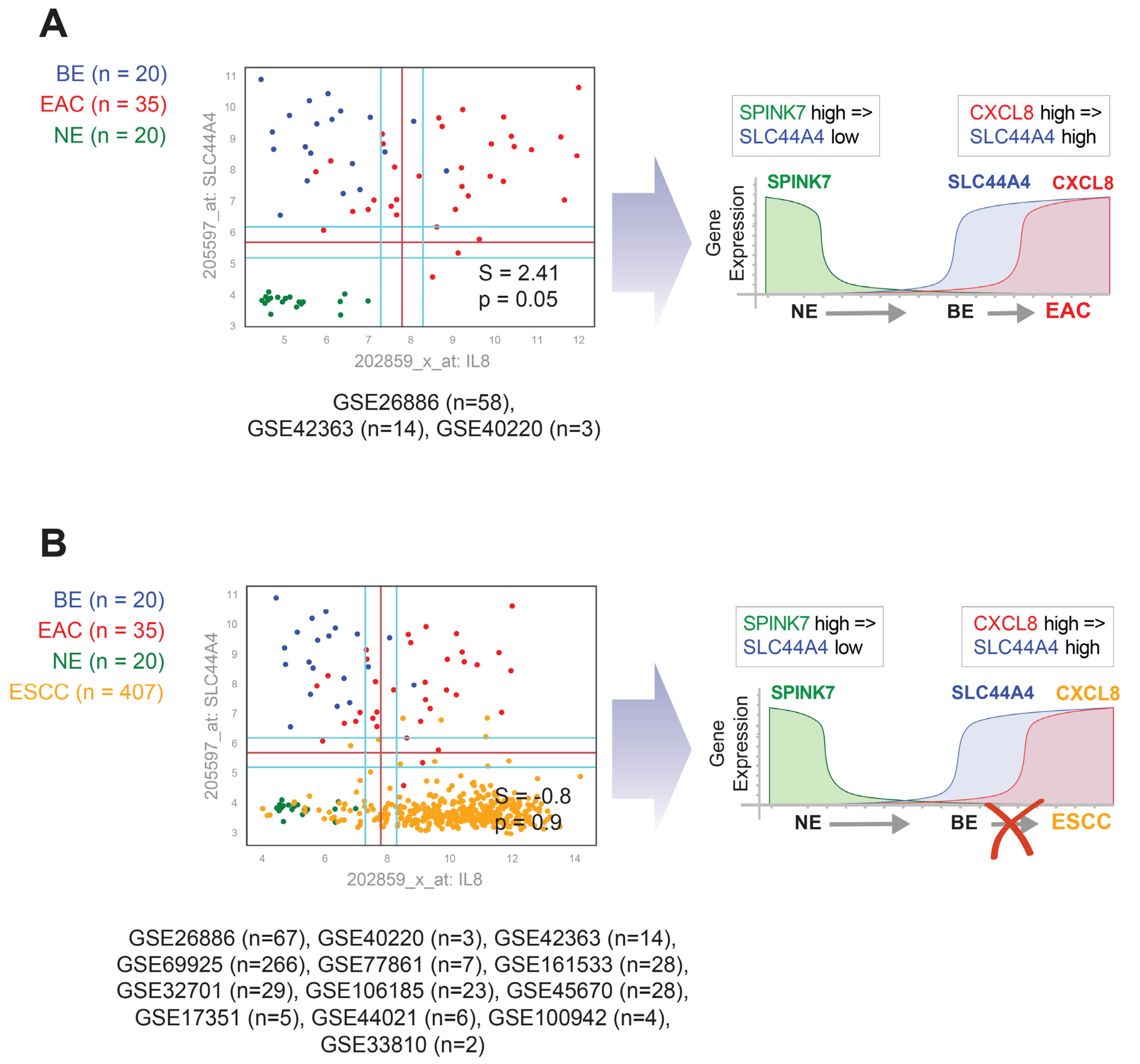

Figure S2. A Boolean logical model of NE→BE→EAC progression shows that EACs, but not ESCCs arise from BE. [Related to Figure 4]

A. A scatterplot (left) for *CXCL8* and *SLC44A4* expression in a pooled cohort of dataset (individual GSEID# are indicated beneath the graph). All of them were microarray datasets generated using the [HG-U133_Plus_2] Affymetrix Human Genome U133 Plus 2.0 Array GPL570 platform). *CXCL8* high => *SLC44A4* high (S = 2.41, p = 0.05, FDR < 0.001) in an invariant Boolean implication relationship in NE, BE and EAC samples where each sample type is mostly confined to one quadrant (NE, bottom-left; BE, top-left; and EAC, top-right). Schematic (right) to visualize the mathematical model of NE→BE→EAC progression based on MiDReG[22, 23] analysis using Boolean Implication relationships. Since *CXCL8* high => *SLC44A4* high, and both *CXCL8* and *SLC44A4* are low in NE, the invariant model suggests that BE stage (*SLC44A4* high, *CXCL8* low) must precede *CXCL8* high and *SLC44A4* high.

B. A scatterplot (left) generated as in A in a pooled cohort of dataset to include ESCCs (individual GSEID# are indicated beneath the graph). Unlike EACs (A), ESCCs fail to remain confined to the upper right quadrant. All of them were microarray datasets generated using the [HG-U133_Plus_2] Affymetrix Human Genome U133 Plus 2.0 Array GPL570 platform). Schematic (right) to visualize the conclusion of the scatterplot, showing that NE→BE→EAC progression model fails in the case of BE→ESCC progression.

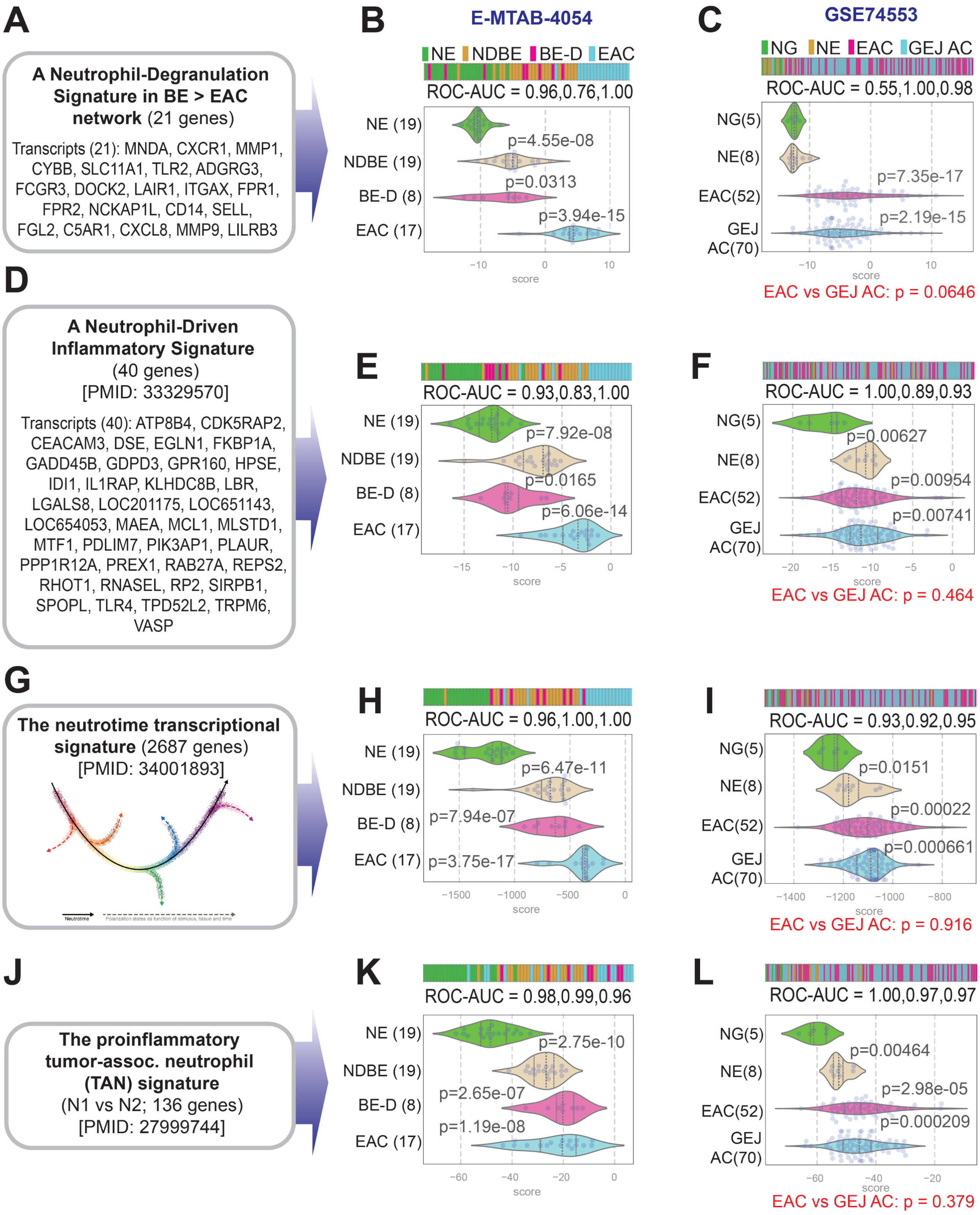

Figure S3. The EAC and GEJ-AC tumor immune microenvironments are similar, and characterized by neutrophil processes [Related to Figure 3]

Bar plots (top) and violin plots (bottom) show the composite scores of upregulated gene clusters (panels A, D, J, E) in normal esophagus (NE), non-dysplastic BE (NDBE), dysplastic BE (DBE), and EACs in E-MTAB-4045 (B, E, H, K) and normal gastric (NG), normal esophagus (NE), EAC and GEJ-AC in GSE74553 (C, F, I, L). *P* values indicate comparison of each sample type against the NE (middle) or NG (right) samples, as determined by Welch's t-test. The neutrophil degranulation (ND) signature (A) was derived from the EAC signatures identified in this work.

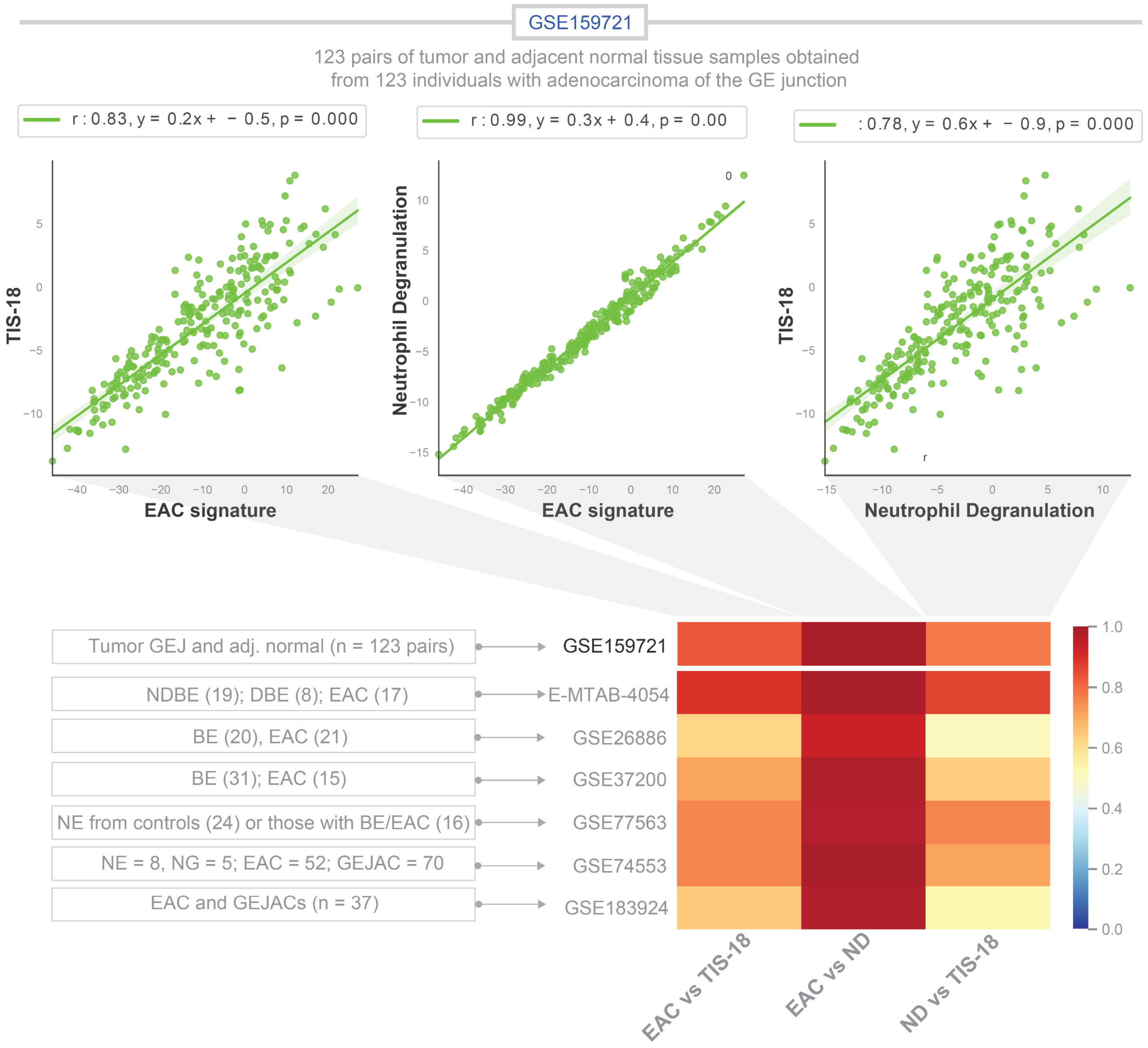

Figure S4. The neutrophil degranulation signature in EACs and GEJ-ACs correlate positively with tumor inflammatory signature (TIS) which is indicative of benefit from immune checkpoint therapy. [Related to Figure 3]

Top: Correlation tests were calculated in GSE159721 (123 pairs of normal GEJ and GEJ-ACs) and displayed as scatter plots using python seaborn lmplots with the p-values between the 18-gene TIS signature on the Y axis and EAC signature on the X axis (*left*), neutrophil degranulation signature on the Y axis and the EAC signature on the X axis (*middle*) and the 18-gene TIS signature on the Y axis and neutrophil degranulation signature in the X axis (*right*).

Bottom: “r” values from correlation tests calculated on numerous independent cohorts are displayed as heatmap.

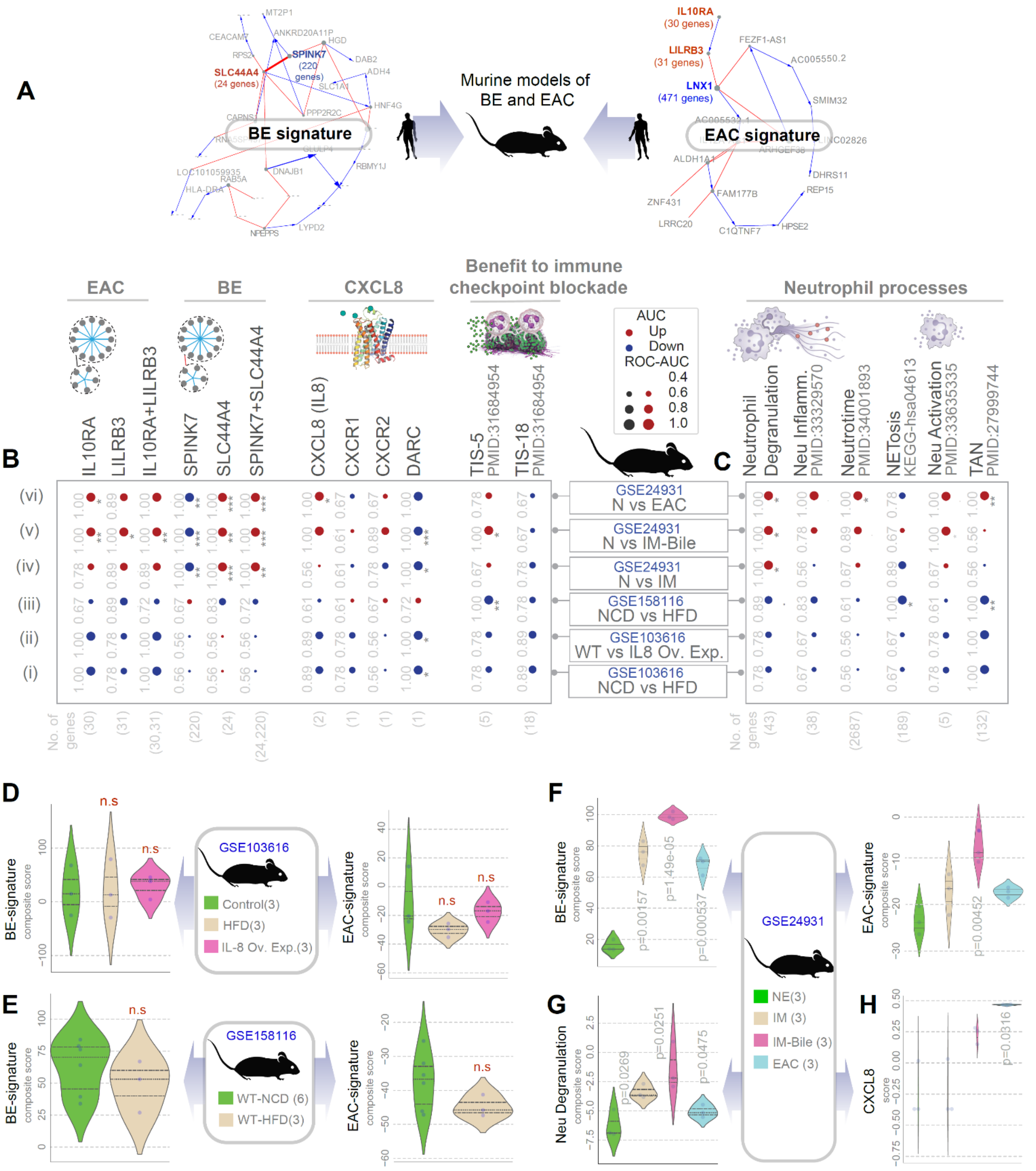

Figure S5. The tumor immune microenvironment in human EAC is rarely recapitulated in murine EAC models [Related to Figure 4]

A. Schematic displays the general strategy used here to vet the appropriateness of murine models for modeling human diseases using human Boolean map-derived BE/EAC signatures. Gene signatures from these human maps are assessed for induction in the murine datasets.

B-C. Murine BE-EAC immune microenvironment was analyzed using the same set of signatures and visualized as bubble plots exactly as in **Figure 4F-G**. IM, intestinal metaplasia. NCD, normal chow diet; HFD, high fat diet; Ov Exp, overexpression. Violin plots for selected signatures are displayed in **C-F.**

C-F. Violin plots showing the composite scores of upregulated gene clusters (BE, EAC, Neu degranulation) or single gene (*CXCL8*) analyzed in murine models of BE→EAC progression, which includes high fat diet (HFD; GSE103616; GSE158116) and IL8-overexpression (Ov. Exp.; GSE103616. *P* values assessed using Welch's test. n.s., not significant. NE, normal esophagus; IM = intestinal metaplasia.

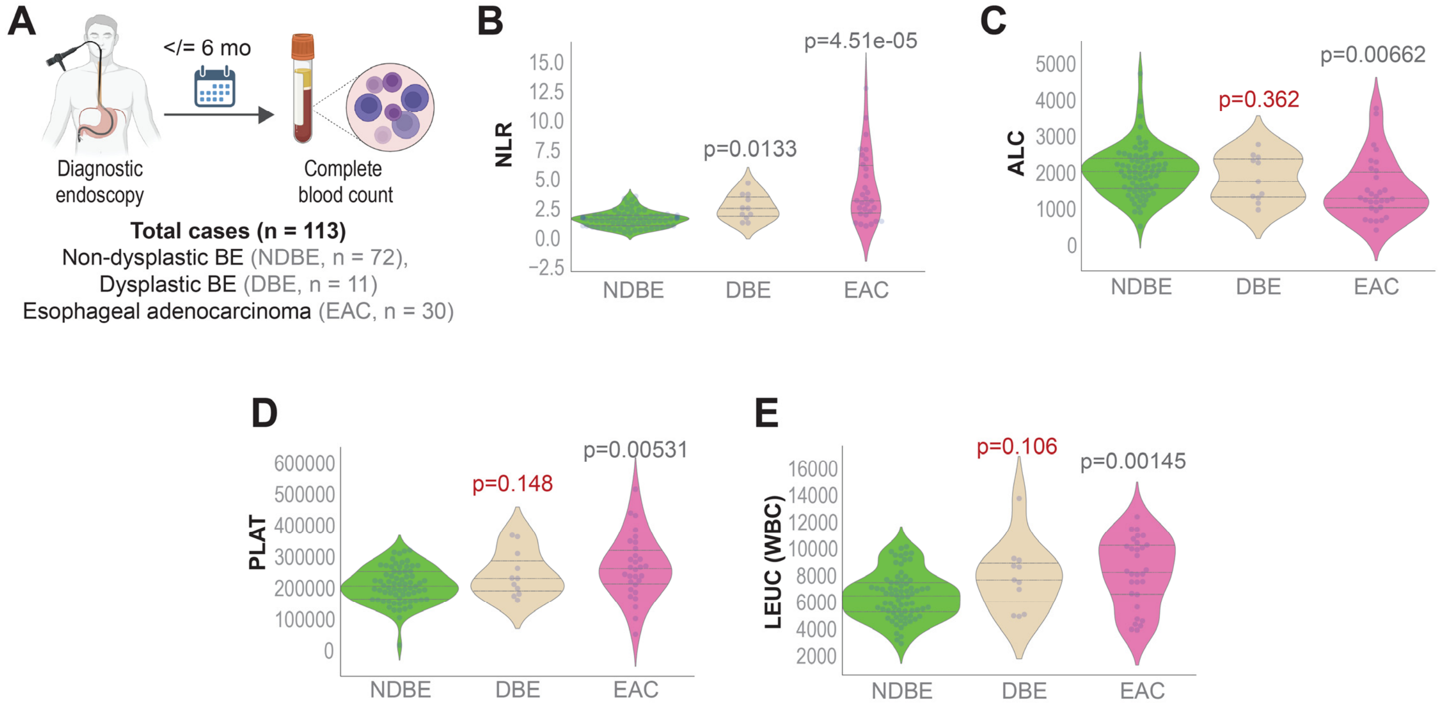

Figure S6. Peripheral neutrophilia and signatures of tumor neutrophil infiltration prognosticate risk of EAC progression. [Related to Figure 5]

A. Schematic summarizing the cohort composition of a cross-sectional study that is analyzed in panels B-E.

B. Violin plots display the neutrophil:lymphocyte ratio (NLR, B), or absolute lymphocyte (ALC, C), platelet (PLAT, D) and leukocyte (LEUK, E) counts in various patients within each diagnostic group shown in panel A. *P* values indicate comparison of each subgroup against the non-dysplastic BE (NDBE) group, as determined by Welch's t-test.

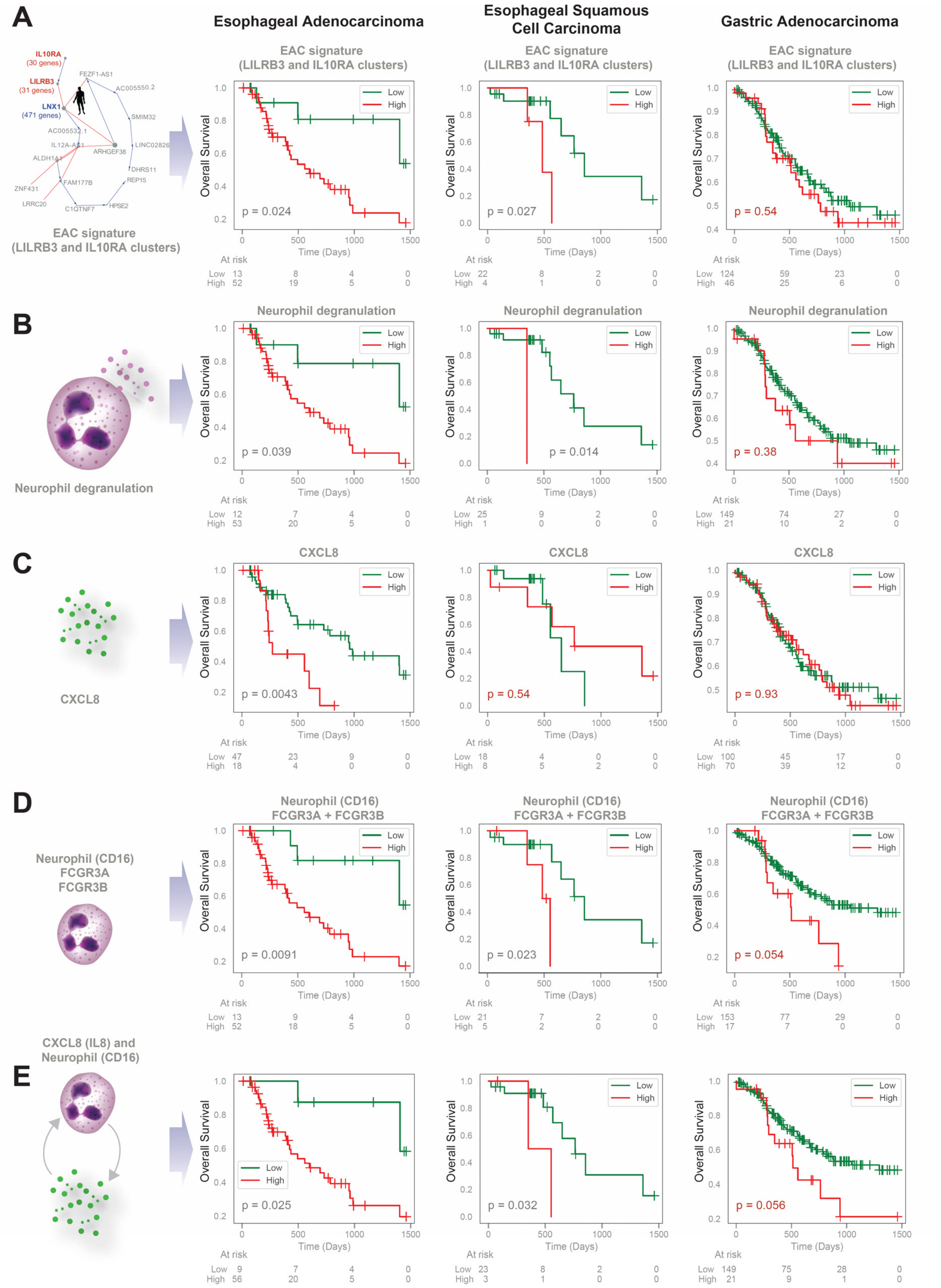

Figure S7. Prognostic value of gene signatures in EACs, ESCCs and gastric adenocarcinomas (GCs). [Related to Figure 5]

Kaplan-Meier (KM) plots display the overall survival of patients with tumors stratified based on the high vs low composite scores of gene signatures displayed as schematics on the left. P values were determined by logrank analysis. EAC and ESCC KM plots in panels C and D are repeated from **Figure 5E-F**.

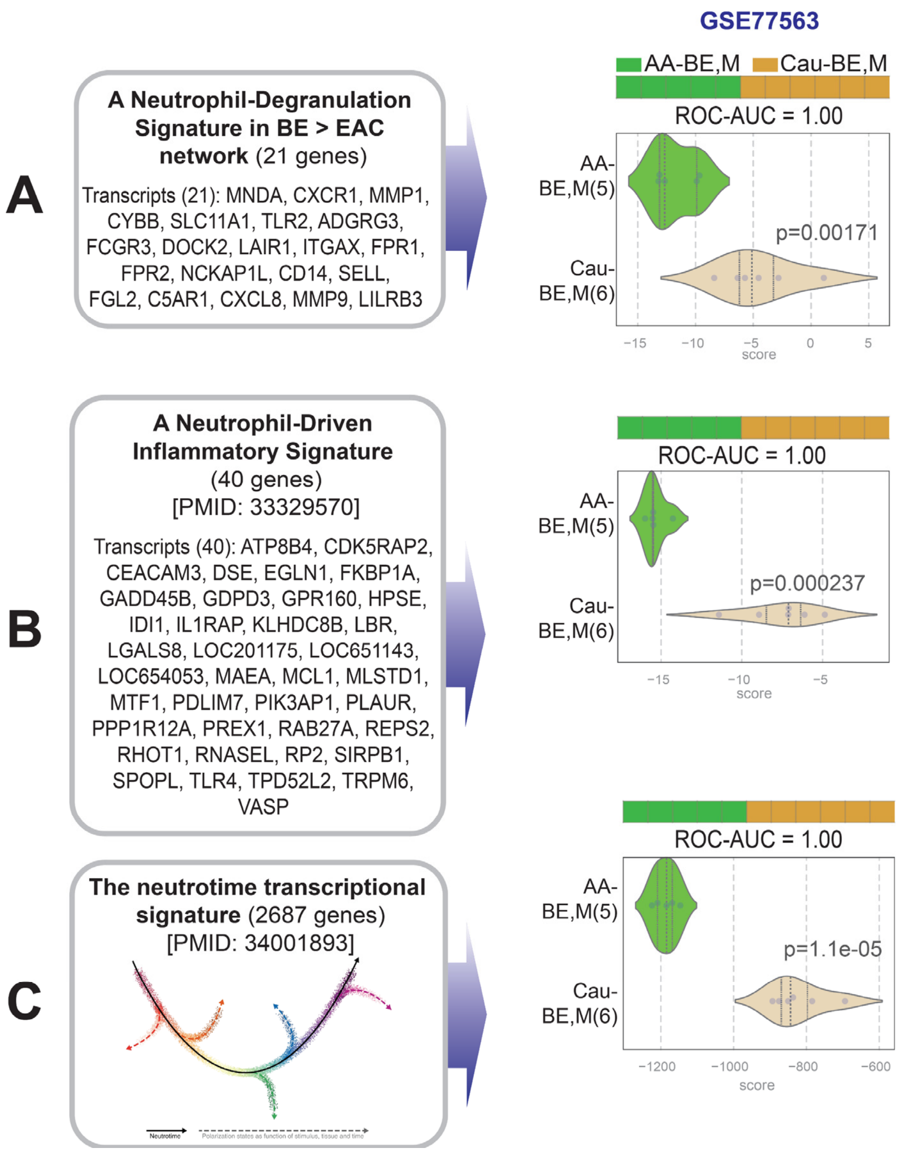

Figure S8. Neutrophil signatures are differentially induced in the histologically normal esophageal lining proximal to BE/EAC lesions in AA vs Cau subjects. [Related to Figure 6]

Bar plots (top) and violin plots (bottom) show the composite scores of upregulated gene clusters (panels A-C) in biopsies from histologically normal esophagus from AA vs Cau subjects with the diagnosis of BE/EAC (in GSE77563). Statistical significance was determined by Welch's t-test. The neutrophil degranulation (ND) signature (A) was derived from the EAC signatures identified in this work.

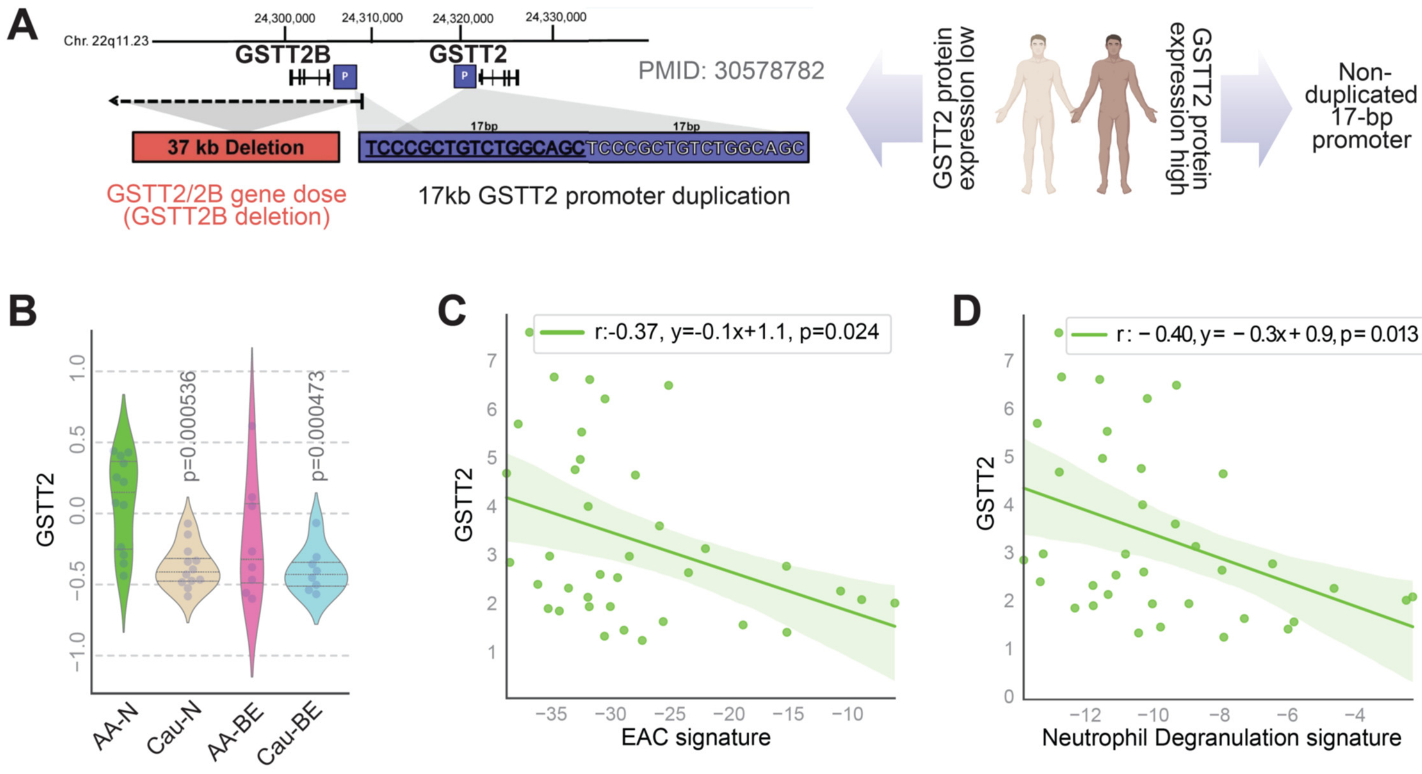

Figure S9. GSTT2 expression inversely correlates with EAC and neutrophil degranulation signatures in the histologically normal esophageal lining proximal to BE/EAC lesions in AA vs Cau subjects. [Related to Figure 6]

A. Schematic summarizing the key conclusions drawn in the only other racially influenced determinant of EAC risk in AA vs Cau. Expression of GSTT2, which protects esophageal squamous cells against DNA damage from genotoxic stress is reduced in Cau compared to AA (right) due to genomic variants at the GSTT2 locus, i.e., either a 37-kb deletion and a 17-bp promoter duplication (left).

B. Violin plots showing the abundance of GTTT2 in normal esophagus from control (AA-N and Cau-N) BE/EACs (AA-BE and Cau-BE) subjects. *P* values indicate comparison of each sample type against the normal samples, as determined by Welch's t-test.

C-D. Correlation tests between GSTT2 on the Y axis and EAC (G) or neutrophil degranulation (H) gene signature scores on the X axis were calculated and displayed as scatter plots using python seaborn lmplots with the p-values. The confidence interval around the regression line is indicated with shades.

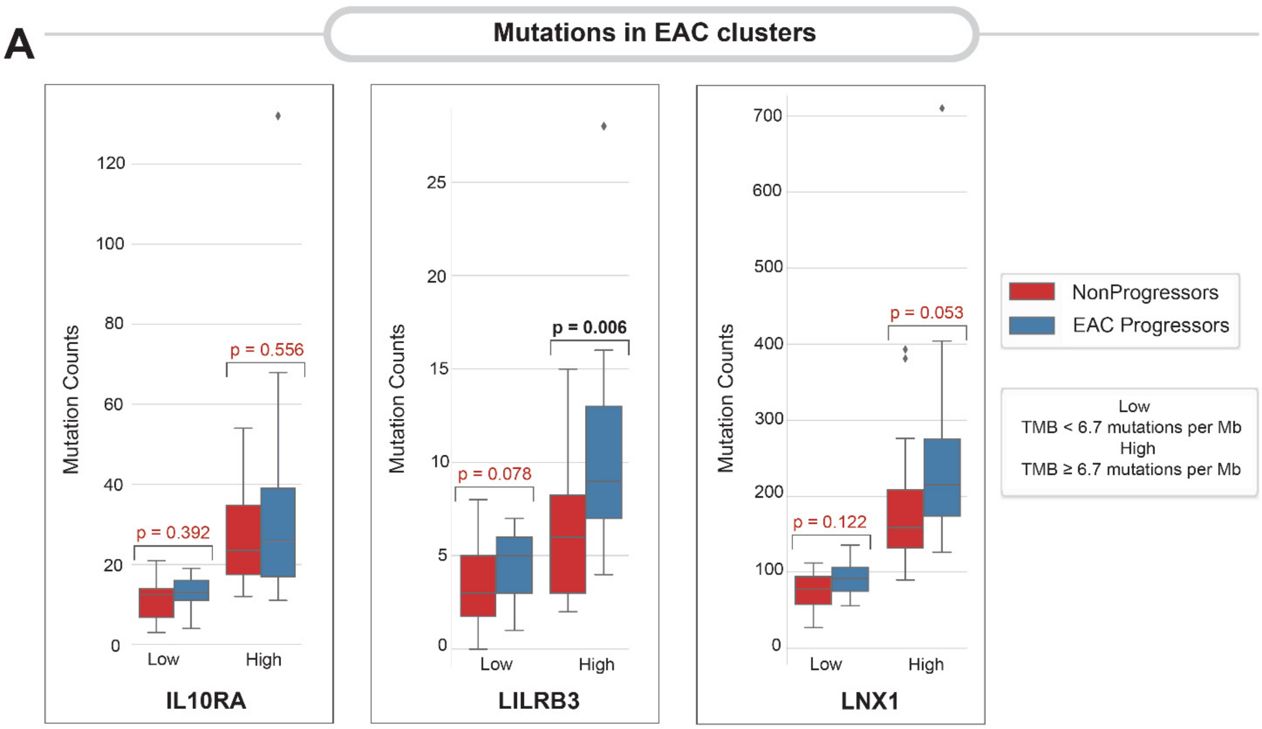

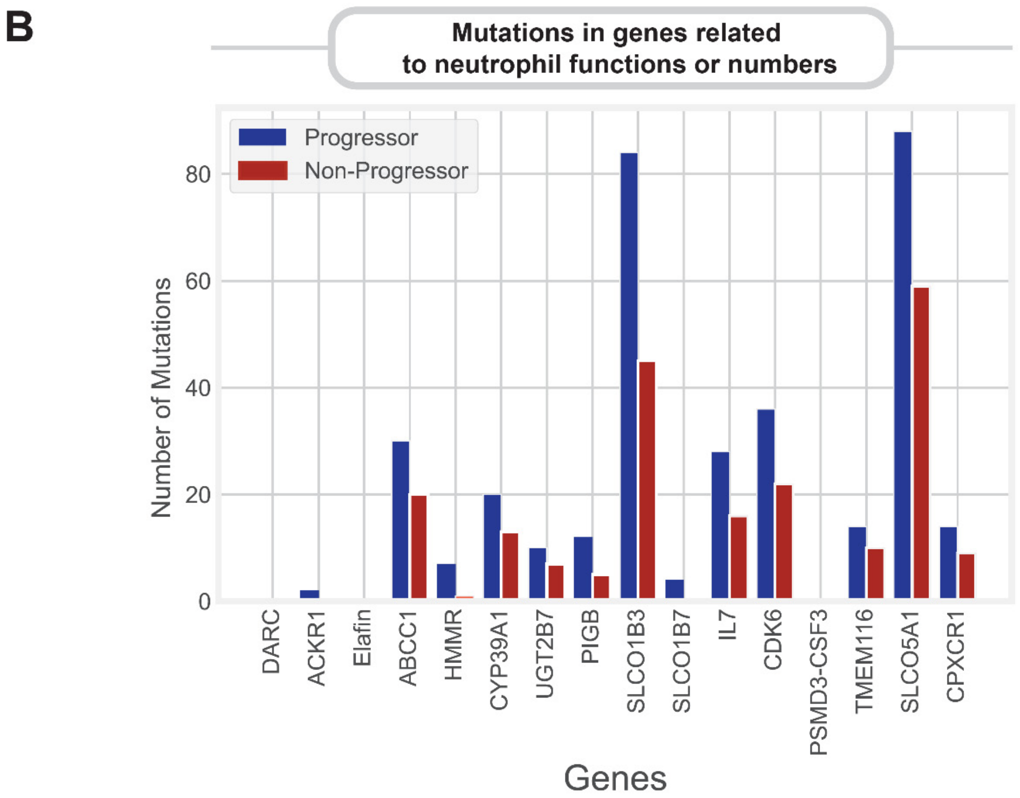

Figure S10. Somatic mutations in genes within the EAC signature or those related to neutrophil function. [Related to Figure 7]

A. Mutational counts in invariant gene clusters. Numbers of somatic mutations detected in the 3 gene clusters (identified in the EAC map) in BE patients who progressed to EAC ('EAC Progressors', n=40) and age-matched BE patients who did not ('Non-Progressors', n=40) [data from[50]]. P-values from two-sided Mann–Whitney *U*-tests are reported above each plot comparing Progressor and Non-progressor patients for each cluster.

B. Somatic mutations in neutrophil genes. Total mutation counts in neutrophil function associated genes across all Progressor patients (blue bars) vs all non-progressor patients (red bars) [data from[50]]. Consensus mutations required to be called by 4 variant callers (MuTect2, VarScan, Strelka, MuSe).

REFERENCES CITED:

1. Sahoo, D. et al. Artificial intelligence guided discovery of a barrier protective therapy in inflammatory bowel disease. *Nat Commun* (2021).
2. Cummings, L.C. et al. A nonrandomized trial of vitamin D supplementation for Barrett's esophagus. *PLoS One* **12**, e0184928 (2017).
3. Hyland, P.L. et al. Global changes in gene expression of Barrett's esophagus compared to normal squamous esophagus and gastric cardia tissues. *PLoS One* **9**, e93219 (2014).
4. Azouz, N.P. et al. The antiprotease SPINK7 serves as an inhibitory checkpoint for esophageal epithelial inflammatory responses. *Sci Transl Med* **10** (2018).
5. Nabokina, S.M., Ramos, M.B., Valle, J.E. & Said, H.M. Regulation of basal promoter activity of the human thiamine pyrophosphate transporter SLC44A4 in human intestinal epithelial cells. *Am J Physiol Cell Physiol* **308**, C750-757 (2015).
6. Redston, M. et al. Abnormal TP53 Predicts Risk of Progression in Patients With Barrett's Esophagus Regardless of a Diagnosis of Dysplasia. *Gastroenterology* (2021).
7. Kastelein, F. et al. Aberrant p53 protein expression is associated with an increased risk of neoplastic progression in patients with Barrett's oesophagus. *Gut* **62**, 1676-1683 (2013).
8. Snyder, P. et al. Aberrant p53 Immunostaining in Barrett's Esophagus Predicts Neoplastic Progression: Systematic Review and Meta-Analyses. *Dig Dis Sci* **64**, 1089-1097 (2019).
9. Bailey, T. et al. Altered cadherin and catenin complexes in the Barrett's esophagus-dysplasia-adenocarcinoma sequence: correlation with disease progression and dedifferentiation. *Am J Pathol* **152**, 135-144 (1998).
10. Suchorolski, M.T., Paulson, T.G., Sanchez, C.A., Hockenbery, D. & Reid, B.J. Warburg and Crabtree effects in premalignant Barrett's esophagus cell lines with active mitochondria. *PLoS One* **8**, e56884 (2013).
11. Of men, not mice. *Nat Med* **19**, 379 (2013).
12. Hellman, S. & Hellman, D.S. Of mice but not men. Problems of the randomized clinical trial. *N Engl J Med* **324**, 1585-1589 (1991).
13. Warren, H.S. et al. Mice are not men. *Proc Natl Acad Sci U S A* **112**, E345 (2015).
14. Mestas, J. & Hughes, C.C. Of mice and not men: differences between mouse and human immunology. *J Immunol* **172**, 2731-2738 (2004).
15. Seok, J. et al. Genomic responses in mouse models poorly mimic human inflammatory diseases. *Proc Natl Acad Sci U S A* **110**, 3507-3512 (2013).
16. Kunze, B. et al. Notch signaling drives development of Barrett's metaplasia from Dclk1-positive epithelial tuft cells in the murine gastric mucosa. *Sci Rep* **11**, 4509 (2021).
17. Kunze, B. et al. Notch Signaling Mediates Differentiation in Barrett's Esophagus and Promotes Progression to Adenocarcinoma. *Gastroenterology* **159**, 575-590 (2020).
18. Munch, N.S. et al. High-Fat Diet Accelerates Carcinogenesis in a Mouse Model of Barrett's Esophagus via Interleukin 8 and Alterations to the Gut Microbiome. *Gastroenterology* **157**, 492-506 e492 (2019).
19. Quante, M. et al. Bile acid and inflammation activate gastric cardia stem cells in a mouse model of Barrett-like metaplasia. *Cancer Cell* **21**, 36-51 (2012).
20. Matsuzaki, J. et al. Bile acids increase levels of microRNAs 221 and 222, leading to degradation of CDX2 during esophageal carcinogenesis. *Gastroenterology* **145**, 1300-1311 (2013).
21. Sahoo, D., Dill, D.L., Gentles, A.J., Tibshirani, R. & Plevritis, S.K. Boolean implication networks derived from large scale, whole genome microarray datasets. *Genome Biol* **9**, R157 (2008).
22. Sahoo, D. et al. MiDReG: a method of mining developmentally regulated genes using Boolean implications. *Proc Natl Acad Sci U S A* **107**, 5732-5737 (2010).
23. Inlay, M.A. et al. Ly6d marks the earliest stage of B-cell specification and identifies the branchpoint between B-cell and T-cell development. *Genes Dev* **23**, 2376-2381 (2009).
24. Chen, J.Y. et al. Hoxb5 marks long-term haematopoietic stem cells and reveals a homogenous perivascular niche. *Nature* **530**, 223-227 (2016).
25. Chan, C.K. et al. Identification and specification of the mouse skeletal stem cell. *Cell* **160**, 285-298 (2015).
26. Dimov, I.K. et al. Discriminating cellular heterogeneity using microwell-based RNA cytometry. *Nat Commun* **5**, 3451 (2014).
27. Chan, C.K. et al. Clonal precursor of bone, cartilage, and hematopoietic niche stromal cells. *Proc Natl Acad Sci U S A* **110**, 12643-12648 (2013).

28. Seita, J. et al. Gene Expression Commons: an open platform for absolute gene expression profiling. *PLoS One* **7**, e40321 (2012).
29. Dalerba, P. et al. Single-cell dissection of transcriptional heterogeneity in human colon tumors. *Nat Biotechnol* **29**, 1120-1127 (2011).
30. Dalerba, P. et al. CDX2 as a Prognostic Biomarker in Stage II and Stage III Colon Cancer. *N Engl J Med* **374**, 211-222 (2016).
31. Cheah, M.T. et al. CD14-expressing cancer cells establish the inflammatory and proliferative tumor microenvironment in bladder cancer. *Proc Natl Acad Sci U S A* **112**, 4725-4730 (2015).
32. Volkmer, J.P. et al. Three differentiation states risk-stratify bladder cancer into distinct subtypes. *Proc Natl Acad Sci U S A* **109**, 2078-2083 (2012).
33. Shin, K. et al. Hedgehog signaling restrains bladder cancer progression by eliciting stromal production of urothelial differentiation factors. *Cancer Cell* **26**, 521-533 (2014).
34. Sin, M.L.Y. et al. Deep Sequencing of Urinary RNAs for Bladder Cancer Molecular Diagnostics. *Clin Cancer Res* **23**, 3700-3710 (2017).
35. Sahoo, D. et al. Boolean analysis identifies CD38 as a biomarker of aggressive localized prostate cancer. *Oncotarget* **9**, 6550-6561 (2018).
36. Bhamre, S., Sahoo, D., Tibshirani, R., Dill, D.L. & Brooks, J.D. Temporal changes in gene expression induced by sulforaphane in human prostate cancer cells. *Prostate* **69**, 181-190 (2009).
37. Sahoo, D. et al. AI-guided discovery of the invariant host response to viral pandemics. *EBioMedicine* **68**, 103390 (2021).
38. Dabydeen, S.A., Desai, A. & Sahoo, D. Unbiased Boolean analysis of public gene expression data for cell cycle gene identification. *Mol Biol Cell* **30**, 1770-1779 (2019).
39. Dang, D. et al. Computational Approach to Identifying Universal Macrophage Biomarkers. *Frontiers in Physiology* **11** (2020).
40. Sahoo, D. et al. Artificial intelligence guided discovery of a barrier-protective therapy in inflammatory bowel disease. *Nat Commun* **12**, 4246 (2021).
41. Edgar, R., Domrachev, M. & Lash, A.E. Gene Expression Omnibus: NCBI gene expression and hybridization array data repository. *Nucleic Acids Res* **30**, 207-210 (2002).
42. Barrett, T. et al. NCBI GEO: archive for functional genomics data sets--update. *Nucleic Acids Res* **41**, D991-995 (2013).
43. Barrett, T. et al. NCBI GEO: mining tens of millions of expression profiles--database and tools update. *Nucleic Acids Res* **35**, D760-765 (2007).
44. Peters, L.A. et al. A functional genomics predictive network model identifies regulators of inflammatory bowel disease. *Nat Genet* **49**, 1437-1449 (2017).
45. Arijs, I. et al. Mucosal gene expression of antimicrobial peptides in inflammatory bowel disease before and after first infliximab treatment. *PLoS One* **4**, e7984 (2009).
46. Sahoo, D., Dill, D.L., Tibshirani, R. & Plevritis, S.K. Extracting binary signals from microarray time-course data. *Nucleic Acids Res* **35**, 3705-3712 (2007).
47. Fabregat, A. et al. The Reactome Pathway Knowledgebase. *Nucleic Acids Res* **46**, D649-D655 (2018).
48. Irizarry, R.A. et al. Summaries of Affymetrix GeneChip probe level data. *Nucleic Acids Res* **31**, e15 (2003).
49. Campos, V.J., Mazzini, G.S., Juchem, J.F. & Gurski, R.R. Neutrophil-Lymphocyte Ratio as a Marker of Progression from Non-Dysplastic Barrett's Esophagus to Esophageal Adenocarcinoma: a Cross-Sectional Retrospective Study. *J Gastrointest Surg* **24**, 8-18 (2020).
50. Thomas, P. et al. *Research Square* (2021).
51. Varghese, F., Bukhari, A.B., Malhotra, R. & De, A. IHC Profiler: an open source plugin for the quantitative evaluation and automated scoring of immunohistochemistry images of human tissue samples. *PLoS One* **9**, e96801 (2014).
52. Li, J. et al. SPT6 promotes epidermal differentiation and blockade of an intestinal-like phenotype through control of transcriptional elongation. *Nat Commun* **12**, 784 (2021).
